# Supplementary material for: Updating age-specific contact structures to match evolving demography in a dynamic mathematical model of tuberculosis vaccination
Source: PLoS Comput Biol. 2022 Apr 22;18(4):e1010002. doi: 10.1371/journal.pcbi.1010002 (PMC9067655; doi:10.1371/journal.pcbi.1010002)
Supplement: S1 Text — Fig A. Model diagram. Fig B. Contact matrix update analysis. Fig C. MCMC chains—M0. Fig D. MCMC chains—M1. Fig E. MCMC chains—M2. Fig F. MCMC chains—M3. Fig G. Posterior distributions of model parameters. Fig H. Calibration and Baseline Projections—M1. Fig I. Calibration and Baseline Projections—M0. Fig J. Calibration and Baseline Projections—M2. Fig K. Calibration and Baseline Projections—M3. Fig L. Model demography. Fig M. Disaggregated TB Incidence. Fig N. Comparison: Prem et al vs unadjusted POLYMOD. Table A. Model Parameters. Table B. Calibration Targets. Table C. Vaccine impact. (PDF) [file pcbi.1010002.s001.pdf]

Updating Contact Structures to Match Evolving Demography in a Dynamic Mathematical Model of Tuberculosis Vaccination

Technical Appendix

Chathika K Weerasuriya[1,*], Rebecca C Harris[1,2], C Finn McQuaid[1], Gabriela B Gomez[3,4], and Richard G White[1]

[1]TB Modelling Group, TB Centre and Centre for the Mathematical Modelling of Infectious Diseases, Department of Infectious Disease Epidemiology, Faculty of Epidemiology & Population Health, London School of Hygiene and Tropical Medicine, London WC1E 7HT, UK
[2]Current address: COVID-19 Medical Franchise, Sanofi Pasteur, Singapore 189767, Singapore
[3]Department of Global Health & Development, Faculty of Public Health & Policy, London School of Hygiene and Tropical Medicine, London, WC1E 7HT, UK
[4]Current address: Department of Modelling, Epidemiology and Data Sciences, Sanofi Pasteur, 69007 Lyon, France
[*]Correspondence: c.weerasuriya@lshtm.ac.uk

Contents

A. Natural History Model **3**
A.1. Demography . . . 3

B. Parameterisation **5**
B.1. Tuberculosis Transmission . . . 5
B.2. Contact Matrix Update Methods . . . 6
B.3. Model Parameterisation . . . 8
B.4. Model Equations . . . 10
B.5. Vaccine Implementation . . . 12

C. Calibration **13**
C.1. Targets . . . 13
C.2. Methods . . . 13

D. Calibration and Baseline Projections **15**
D.1. ABC-MCMC Sampler Performance . . . 15
D.2. Posterior Distributions . . . 15
D.3. Baseline Projections . . . 21
D.3.1. Model Demography . . . 21
D.3.2. Epidemic Analysis . . . 27

E. Vaccine Impact **29**

F. Country Adjusted Matrix **29**

References **31**

Supplementary Methods

A. Natural History Model

We constructed a difference equation-based compartmental dynamic transmission model of *Mycobacterium tuberculosis* (Mtb) , with a timestep of 6 months and a time horizon of 1950-2050. The model was an evolution of previous compartmental models developed and used by Knight et al. [1] , Harris et al. [2, 3], and Weerasuriya et al. [4, 5]. The model had the following compartments:

1. Susceptible (S; never infected by Mtb);
2. Latently infected (L; infected with Mtb, but without active disease);
3. Infectious active disease (I; bacteriologically positive TB disease capable of transmission);
4. Non-infectious active disease (N; bacteriologically negative TB and incapable of transmission);
5. On-treatment for tuberculosis (T) and;
6. Resolved (R; recovered from active disease, either via treatment or through natural cure).

A diagram of model compartments and flows between compartments is presented in Figure A.

Susceptible (naive) populations infected by Mtb could either become latently infected or directly progress to active disease. Individuals in the active disease state could either: (1) be detected and move to treatment; (2) cure TB disease naturally; or (3) die from tuberculosis or other causes. Those who experienced natural cure moved to the resolved compartment. Those starting treatments entered the treatment state where they could experience either treatment success (and move to the resolved state) or treatment failure (and return to noninfectious active disease). Latently infected and resolved populations could reactivate and relapse, respectively, back into the active disease state. Alternatively, these populations could be reinfected and experience rapid progression to active disease. We applied background age-specific mortality to all compartments.

A.1. Demography

The model was structured into three age classes: children (C; < 15 years), adults (A; ≥ 15 and < 65 years) and elderly (E; ≥ 65 and ≤ 99 years). New births entered the chidren S compartment at the beginning of each year. Annual mortality and ageing rates out of each age class were derived from the United Nations World Population Prospects [6] for India, using the medium estimates of projected population from 2019–2050. UN population estimates and projections of all-cause mortality include TB mortality. During

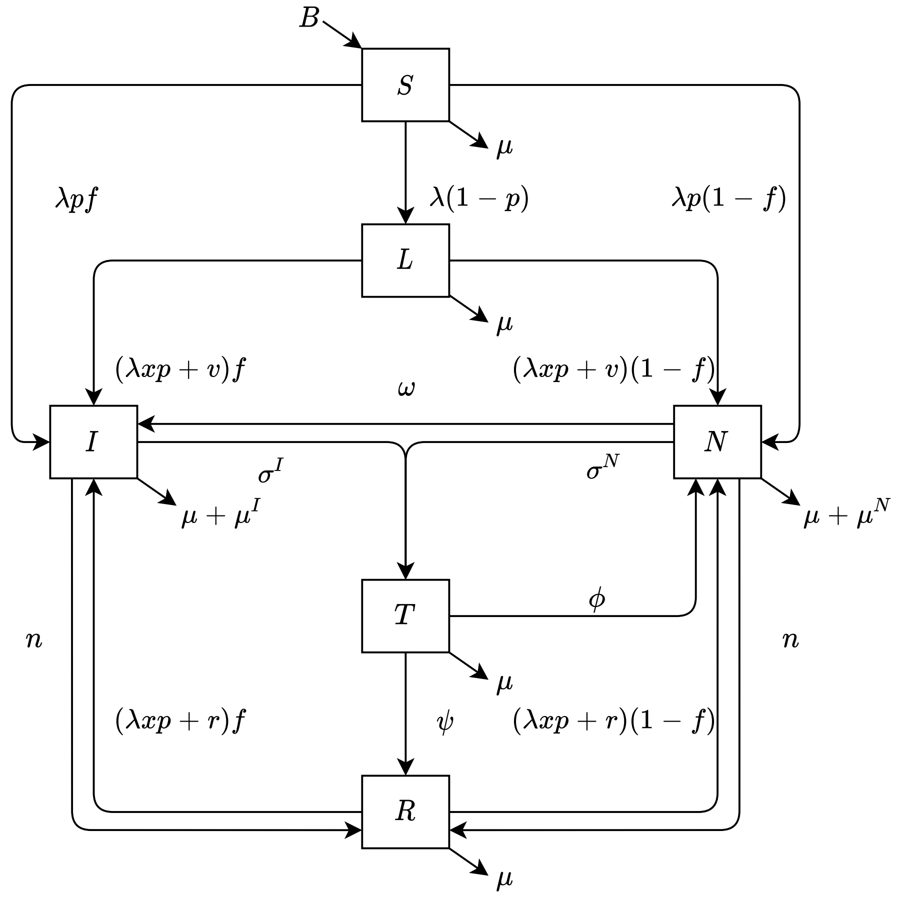

Figure A: Model diagram. Key for symbols given in Table A. Boxes represent model compartments, arrows represent transitions. Transitions B and μ represent births into the model and non-TB background mortality, respectively.

model calibration (section §C), we removed model-estimated TB deaths from background mortality and recalculated cause-deleted background mortality. Cause-deleted background mortality was stored for each calibrated parameter set and used as the model input during vaccine simulation (section B.5).

B. Parameterisation

Prior ranges, references, and constraints for model parameters are presented in Table A. Mtb transmission was modelled using the force of infection parameter λ section B.1. Following infection by Mtb, susceptible individuals transitioned directly to active disease ("fast-progression") or latency with age-specific risk p and $1-p$ respectively. Fast-progressors were divided into infectious vs noninfectious active disease via age-specific parameters f and $1-f$, respectively. Individuals in the L and R compartments could reactivate or relapse, respectively, per age-specific risk parameters v and r. Latently infected individuals and individuals in the recovered compartment could be reinfected and progress to active disease. However current or previous infection was assumed to confer protection against fast-progression to disease: risk of disease following reinfection was reduced per parameter x (where $1-x$ equaled the relative degree of protection). Reinfected latently infected individuals who did not fast-progress remained in the L compartment. However, as relapse rates from recovered are higher than reactivation rates from latency [7, 8, 9], reinfected recovered individuals who did not fast-progress did not become latently infected. This would have implied that reinfection would reduce the risk of active disease (from r to v) in recovered individuals, which we considered implausible.

B.1. Tuberculosis Transmission

We adapted the age-specific force of infection formulation, λ_i from Keeling and Rohani [10] for a difference equation model. This formulation assumed some probability π of infection following an infectious contact; normalised average daily contact rate κ (section B.2), multiplied by 180 to scale to a six-month time step; proportion I of infectious individuals in the population (i.e. bacteriologically-positive TB prevalence) and δq representing the probability of infection in some small time interval δt. Assuming each contact is an independent event, the probability of avoiding infection in some interval δt was

$$1 - \delta q = (1-\pi)^{180\kappa I \delta t}$$

leading to the expression for the probability of infection in time period δt:

$$\delta q = 1 - (1-\pi)^{180\kappa I \delta t}$$

We took the scaled effective contact rate, β, to as:

$$\beta = -180\kappa \cdot \ln(1-\pi)$$

We constructed a pairwise matrix (B) containing β terms for all combinations of children (c), adults (a) and the elderly (e):

$$B = \begin{bmatrix} \beta_{c,c} & \beta_{c,a} & \beta_{c,e} \\ \beta_{a,c} & \beta_{a,a} & \beta_{a,e} \\ \beta_{e,c} & \beta_{e,a} & \beta_{e,e} \end{bmatrix} = -180 \cdot \ln(1-\pi) \cdot \begin{bmatrix} \kappa_{c,c} & \kappa_{c,a} & \kappa_{c,e} \\ \kappa_{a,c} & \kappa_{a,a} & \kappa_{a,e} \\ \kappa_{e,c} & \kappa_{e,a} & \kappa_{e,e} \end{bmatrix}$$

We assumed that π was constant across all ages. Thus, differential transmission to age classes was driven solely by heterogeneity in contact rates, represented by matrix κ. Each constituent term in the κ matrix, $\kappa_{i,j}$ represented the number of contacts each member of i made with group j per day, normalised to give an overall population-wide average contact rate of one per day.

The population proportion of age-specific bacteriologically-positive TB prevalence was given by vector I as

$$I = \begin{bmatrix} I_c \\ I_a \\ I_e \end{bmatrix}$$

leading to the age-specific force of infection, λ_i,at time t:

$$\lambda_{i,t} = 1 - \exp(-BI_{t-1})$$

As this study normalised the contact matrix κ to have a population-wide average contact rate of 1 day^{-1}, π represented an aggregate transmission scaling factor comprising a correction for contact rate and transmission probability.

B.2. Contact Matrix Update Methods

We adapted contact matrix transformation methods from Arregui et al. [11]. We first derived the base contact matrix from aggregated POLYMOD [12] data (Fig 1A in the main text). Then, we divided each contact rate by the overall average contact rate of the source population (approximately 12.79 contacts per day) to provide a normalised matrix $\kappa_{i,j}$.

Here we describe the methods to generate a new contact matrix $\kappa'_{i,j}$, matching the demographic specific composition of population N', containing age groups i and j of magnitude N'_i and N'_j, from some initial matrix $\kappa_{i,j}$, originating from a population N, where age groups i and j have magnitudes N_i and N_j, respectively.

M0

The contact matrix was constant at each time step in the model, applying no corrections to match demography. Median reciprocity error—defined as the fraction of non-reciprocal contacts among all contacts between non-self age-group pairs [11]—is presented in figure B:**A**. There was substantial reciprocity error over the model time horizon , including over the vaccine simulation period of 2025 (56%) to 2050 (21%).

The $\mathrm{M0}$ method is computationally simple but fails to preserve reciprocity. This may not introduce substantial error, for example, when modelling annual viral epidemics, or in populations where the demographic composition is stable. However, tuberculosis models routinely project over decades, very likely encountering substantial demographic change.

The effect of unadapted, unbalanced matrices are highlighted by considering a different hypothetical population containing age groups l and m, of magnitude N_l and N_m respectively, with contact rates $\kappa_{l,m}$ between each member of l and group m, and $\kappa_{m,l}$ between each member of m and group l. If N_l is increased to N'_l, holding $\kappa_{l,m}$, $\kappa_{m,l}$, and N_m constant, the total contacts with l, by all members of m, $N_m\kappa_{m,l}$ would be fewer than the total new number of contacts with m by l, $N'_l\kappa_{l,m}$, thus underestimating the force of infection experienced by members of m.

M1

The M1 method avoids the reciprocity error inherent to M0, as $\kappa_{i,j}$ and $\kappa_{j,i}$ are recalculated taking the total contacts as the mean of $N'_i\kappa_{i,j}$ and $N'_j\kappa_{j,i}$. This update was applied at each time step, formulated as:

$$\kappa'_{i,j} = \frac{1}{2N'_i}\left(\kappa_{i,j}N'_i + \kappa_{j,i}N'_j\right)$$

By definition, the contact rate of an age-group with itself does not change.

M2

The contact rate between age groups i and j is a function of both intrinsic preference for contact with j by i, relative to contact with other i (i.e., *assortativity*), and the relative *density* of N_j (i.e., $\frac{N_j}{N_i+N_j}$) in the total population. For every contact matrix Q with n age classes, we can compute a corresponding *assortativity matrix* R. The entries of R are the contact rates between i and j, multiplied by a constant factor n, expected in a population where the n classes are equally sized and where the relative preferences for contact between groups are the same as in Q. We derived the base assortativity matrix, A, from the original POLYMOD matrix as:

$$A_{i,j} = \kappa_{i,j}\frac{N}{N_j}.$$

We then recalculated the updated contact matrix during each time step of the model run,

$$\kappa'_{i,j} = A_{i,j}\frac{N'_j}{N'}.$$

In both M0 and M1, despite the changing density of l from of $\frac{N_l}{N}$ to $\frac{N'_l}{N'}$, the contact rate $\kappa_{l,l}$ remains unchanged, implying a counterbalancing change in assortativity.

Previous studies have quantifed assortativity into an aggregate measure referred to as the *assortativity coefficient* [13], ρ:

$$\rho = \frac{\sum_i \epsilon_{ii} - \sum_i \alpha_i \gamma_i}{1 - \sum_i \alpha_i \gamma_i},$$

where ϵ is a matrix of the fraction of all contacts occuring between each age group pair; α_i is the fraction of all contacts with group i, *by* group i; and γ_i is the fraction of all contacts *by* group i *with* group i. In a balanced contact matrix, $\alpha_i = \gamma_i$.

This coefficient ranges from -1 to $+1$, where $+1$ represents completely assortative mixing (where each group mixes exclusively within itself), 0 represents random (homogeneous) mixing, and a value $\geq -1, < 0$ for completely disassortative mixing. We observed increasing assortativity in the M0 and M1 models over the model time horizon, with ρ between approximately 0.3 and 0.45 (figure B:**B**). Assortativity remained constant at approximately 0.45 for both M2 and M3 models.

M3

In the M3 update method, we normalised the assortativity matrix derived from the M2 model (denoted here as $\hat{\kappa}$) to have a population-wide average contact of one, as:

$$\hat{\kappa}_{i,j} = \kappa_{i,j} \frac{N N'_j}{N_j N'}$$
$$d = \frac{\sum_{i,j} \hat{\kappa}_{i,j} N'_i}{N'}$$
$$\kappa'_{i,j} = \frac{\hat{\kappa}_{i,j}}{d}$$

We found that average contact rate was higher in the M2 than the M1 model over the model time horizon. Average contact in M1 remained stable at approximately 14.5 contacts per day (figure B:**C**).

B.3. Model Parameterisation

Natural history parameters with prior ranges and references are presented in table A.

We stratified parameters for rates of fast progression following infection (p), progression to infectious vs. non-infectious disease (f), reactivation from latency (v), relapse from the recovered state (r), and TB mortality (separately for infectious [μ^{I}] vs non-infectious disease [μ^{N}]) by age for children, adults, and the elderly, independently sampling each from age-specific priors during calibration (table A). There is no empirical data to substantiate the upper bound for fast-progression rate in the elderly; therefore, we used progression rates in HIV+ populations for this value, assuming that immunocompromise was a reasonable proxy for immunosenescence. Where age-specific prior ranges overlapped, we constrained the sampling process to retain values for progression rate, reactivation from latency, and TB mortality, in children and the elderly only if greater than in adults. Similarly, natural cure rate was constrained to be lower in the elderly than adults. We fitted treatment initiation rate ($c2020$) in 2020, interpolating from zero in 1960 to the fitted value in 2020. Treatment outcomes were taken from WHO [14] data.

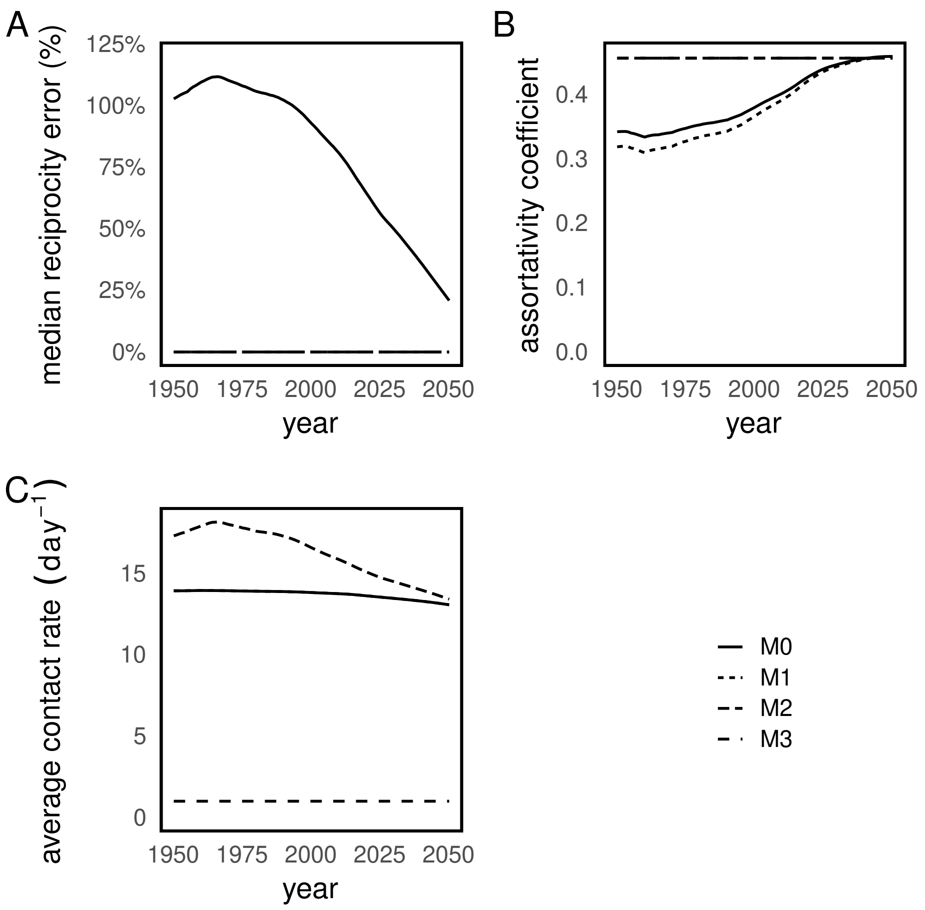

Figure B: Contact matrix update analysis. **A** Median reciprocity error. Lines for M0–M2 are overlaid on one another. **B** Median assortativity coefficient, ρ. Lines for M2 and M3 are overlaid on one another. **C** Median value for average contact rate per day.

Both treatment initiation rates and outcomes were held constant after 2020.

B.4. Model Equations

Equations describing transitions between model compartments are given in this section. In the following equations, subscript t represents the current time step and j represents a generic age class. Symbol definitions are presented in table A. Where indicated, subscripts c, a, and e specifically refer to child, adult, and elderly specific states, respectively. As the model time-step was defined as six months (the duration of first-line therapy for active tuberculosis), outflow from the treatment compartment (T) is the entire content of that compartment in step $t-1$.

Susceptible (S) compartment

$$S_{t,j} = (1 - \lambda_{t-1,j})S_{t-1,j}$$

Latent Infection (L) compartment

$$\begin{aligned} L_{t,j} = {} & (1 - v_j - \lambda_{t-1,j} x p_j - \mu_{t-1,j})L_{t-1,j} \\ & + (1 - p_j)\lambda_{t-1,j} S_{t-1,j} \end{aligned}$$

Active infectious disease (I) compartment

$$\begin{aligned} I_{t,j} = {} & (1 - n_j - \sigma^{\mathrm{I}}_{t-1} - \mu^{\mathrm{I}}_j - \mu_{t-1,j})I_{t-1,j} \\ & + \lambda_{t-1,j} p_j f_j S_{t-1,j} \\ & + (\lambda_{t-1,j} x p_j + v_j) f_j L_{t-1,j} \\ & + N_{t-1,j}\omega \\ & + (\lambda_{t-1,j} x p_j + r_j) f_j R_{t-1,j} \end{aligned}$$

Active noninfectious (N) compartment

$$\begin{aligned} N_{t,j} = {} & (1 - n_j - \sigma^{\mathrm{N}}_{t-1} - \mu^{\mathrm{N}}_j - \mu_{t-1,j} - \omega)N_{t-1,j} \\ & + \lambda_{t-1,j} p_j (1 - f_j) S_{t-1,j} \\ & + (\lambda_{t-1,j} x p_j + v_j)(1 - f_j) L_{t-1,j} \\ & + (\lambda_{t-1,j} x p_j + r_j)(1 - f_j) R_{t-1,j} \\ & + (1 - \mu_{t-1,j})\phi_{t-1} T_{t-1,j} \end{aligned}$$

Treatment (T) compartment

$$T_{t,j} = \sigma^{\mathrm{I}}_{t-1} I_{t-1,j} + \sigma^{\mathrm{N}}_{t-1} N_{t-1,j}$$

Table A: Model Parameters. Subscript j indicates the parameter was age-stratified, with subscripts c, a, and e representing child, adult, and elderly age groups respectively. We assumed uniform prior ranges for all sampled parameters.

**Parameter and symbols**	**Prior ranges and constraints**	**References**
NATURAL HISTORY PARAMETERS		
Risk of progressing directly to active TB following (re-)infection<br>p_j	$0.01 \leq p_c \leq 0.06$<br>$0.08 \leq p_a \leq 0.2$<br>$p_a \leq p_e \leq 0.36$	[1, 8, 15, 7]
Protection from re-infection or developing active TB due to latent infection or recovered state<br>x	$0.25 \leq x \leq 0.4$	[8, 7, 16, 17]
Risk of reactivation from latent infection or recovered state<br>v_j	$0.0001 \leq v_c \leq 0.0003$<br>$0.0001 \leq v_a \leq 0.0003$<br>$v_a \leq v_e \leq 0.04$	[18, 1, 15, 17, 19]
Proportion of new active cases becoming infectious<br>f_j	$0 \leq f_c \leq 0.15$<br>$0.25 \leq f_a \leq 0.75$<br>$0.19 \leq f_e \leq f_a$	[15, 7, 18, 20, 21, 22]
Risk of converting from noninfectious to infectious active disease<br>ω	$0.007 \leq \omega \leq 0.02$	[23]
Risk of natural cure<br>n_j	$0.1 \leq n_c \leq 0.25$<br>$n_c = n_a$<br>$0.1 \leq n_e \leq n_a$	[15, 7]
Risk of relapse from recovered<br>r_j	$0.005 \leq r_c \leq 0.015$<br>$0.005 \leq r_a \leq 0.015$<br>$r_a \leq r_e \leq 0.015$	[24]
Risk of mortality with active infectious TB<br>μ^{I}	$0 \leq \mu_a^{\mathrm{I}} \leq 0.178$<br>$\mu_a^{\mathrm{I}} \leq \mu_c^{\mathrm{I}} \leq 0.178$<br>$\mu_a^{\mathrm{I}} \leq \mu_e^{\mathrm{I}} \leq 0.178$	[25]
Risk of mortality with active noninfectious TB<br>μ^{N}	$0 \leq \mu_a^{\mathrm{N}} \leq 0.034$<br>$\mu_a^{\mathrm{N}} \leq \mu_c^{\mathrm{N}} \leq 0.034$<br>$\mu_a^{\mathrm{N}} \leq \mu_e^{\mathrm{N}} \leq 0.034$	[25]
Transmission calibration factor<br>π	$0 \leq -\log_{10}(\pi) \leq 2$	Fitted.
TREATMENT AND DIAGNOSIS PARAMETERS		
Risk of treatment initiation in 2020<br>$c2020$	$0 \leq c2020 \leq 1$	Fitted.
Relative detection of non-infectious cases<br>e	$0.4 \leq e \leq 0.8$	Fitted.

Recovered (R) compartment

$$
\begin{aligned}
R_{t,j} &= (1 - \lambda_{t-1,j} x p_j - r_j - \mu_{t-1,j}) R_{t-1,j} \\
&+ (I_{t-1,j} + N_{t-1,j}) n_j \\
&+ (1 - \mu_{t-1,j}) \psi_{t-1} T_{t-1,j}
\end{aligned}
$$

Demography

New births into the model were implemented as new entries into the children age group in the first time step of each calendar year.

$S_{t,c} = S_{t,c} + B_t$

We implemented population aging at every time step as a calendar-time specific risk, calculated from World Population Prospects [6] projections, out of the child compartments into the adult compartments, and out of the adult compartments into the elderly compartments. Individuals aging out of the elderly compartment were assumed to experience 100% mortality. Thus, for any particular compartment Y, aging rates g were applied as:

Ageing out of S, L, and I compartments:

$Y_{j,t} = Y_{j,t} - g_{j,t-1} Y_{j,t-1}$

Ageing out of compartment N:

$N_{j,t} = N_{j,t} - g_{j,t-1}(N_{j,t-1} + T_{j,t-1}\phi_{t-1})$

Ageing out of compartment R:

$R_{j,t} = R_{j,t} - g_{j,t-1}(R_{j,t-1} + T_{j,t-1}\psi_{t-1})$

Aging into compartments S, L, and I:

$Y_{a,t} = Y_{a,t} + g_{c,t-1} Y_{c,t-1}$

$Y_{e,t} = Y_{e,t} + g_{a,t-1} Y_{a,t-1}$

Ageing into compartment N:

$N_{a,t} = N_{a,t} + g_{c,t-1}(N_{c,t-1} + T_{c,t-1}\phi_{t-1})$

$N_{e,t} = N_{e,t} + g_{a,t-1}(N_{a,t-1} + T_{a,t-1}\phi_{t-1})$

Ageing into compartment R:

$R_{a,t} = R_{a,t} + g_{c,t-1}(R_{c,t-1} + T_{c,t-1}\psi_{t-1})$

$R_{e,t} = R_{e,t} + g_{a,t-1}(R_{a,t-1} + T_{a,t-1}\psi_{t-1})$

Aging in and out of the N and R compartments had additional terms to represent aging which occurred to those exiting the T compartment. The foregoing demographic transitions were replicated during vaccine simulation in the vaccinated stratum.

B.5. Vaccine Implementation

We implemented vaccination in the model as described elsewhere [4, 5, 2, 3].

We categorised vaccines qualitatively into two dimensions: by host-infection required for efficacy and by preventive effect. We stratified vaccine by the host-infection status required for efficacy: (1) "preinfection" (PRI) vaccines were only effective in susceptible individuals, with no prior history of infection; (2) "postinfection" (PSI) vaccines were effective in those with latent infection and resolved infection; and (3) "pre- and post-infection" (P&PI) vaccines were effective in all three types of host infection status. We

also stratified vaccines to prevent infection (POI), prevent disease (POD), or both (POI/D). To represent the vaccinated population, we replicated each compartment C to create an equivalent vaccinated compartment C_V.

Vaccination at coverage q was represented by transition from the unvaccinated compartment to its corresponding vaccinated equivalent ($C \to C_V$) and waning of protection at rate omega as transition in the opposite direction ($C_V \to C$).

In previous work, using similarly structured models also set in India [4, 5, 3], we have consistently found that vaccine impact behaved relatively linearly with coverage. On this basis, we presume that relative differences in vaccine impact between different contact matrix update methods are likely to be preserved over changes in immunisation coverage; therefore, we have modelled only a single coverage scenario in this study,

C. Calibration

C.1. Targets

The common calibration targets for all models M0–M3 are presented in table B. We calibrated all models to a minimum of eight calibration targets, including prevalence, mortality, incidence and notification rates [14, 26]. As India has not yet reported a nationally representative tuberculosis prevalence survey, we used estimates of bacteriologically-positive prevalence rate derived through pooling subnational estimates as a calibration target for all TB prevalence rate [27]. We derived age-stratified incidence *rates* from overall incidence estimates published by WHO and population estimates from World Population Prospects [6]. Overall mortality rates were obtained form the WHO Tubeculosis Database [14].

We assumed that treatment initiation rate within the model corresponded to published notification rates. We assumed a 20% uncertainty interval around point estimates of notification rate to adjust for potential private sector (unreported) treatment of TB and for loss to follow up between diagnosis and treatment initiation and calibrated rates of treatment initiation (σ) to this range. As described in the main text, we also calibrated models M0, M2, and M3 to all-age TB incidence rate projected by model M1.

C.2. Methods

We calibrated the model independently for each update method (M0–M3).

We calculated a normalised distance ratio, o, using model output t that required calibration to target t_{mid} within range t_{low}–t_{high} as:

$$o = \begin{cases} \frac{t - t_{\text{mid}}}{t_{\text{high}} - t_{\text{mid}}} & \text{if } t \geq t_{\text{mid}} \\ \frac{t_{\text{mid}} - t}{t_{\text{mid}} - t_{\text{low}}} & \text{if } t < t_{\text{mid}} \end{cases}$$

We applied a penalty for model outputs which fell outside the uncertainty bounds as

[1] For models M0, M2, and M3 only

Table B: Calibration Targets. Target values and ranges are specified as per 100,000 population.

**Target**	**Year**	**Age group**	**Value** midpoint	range	**References**
Incidence	2010	All	247	(128–405)	[14, 26]
	2019	Children ($<$ 15y)	90	(55–126)	
		All	193	(132–266)	
		All Adults ($\geq$ 15y)	230	(140–321)	
		Elderly ($\geq$ 65y)	277	(0–622)	
	2050[1]	All	234	(190–271)	M1 model projection.
Mortality	2019	All	33	(30–35)	[14, 26]
Notification	2019	All	190	(152–228)	[14, 26]
Prevalence	2015	All	253	(195–312)	[27]

$$\hat{o} = \begin{cases} o \cdot n & \text{if } o > 1 \\ o & \text{if } o \leq 1 \end{cases},$$

where n equaled the total number of targets to fit.

We then sampled the parameter space consistent with calibration targets using an Approximate Bayesian Computation Markov Chain Monte Carlo (ABC-MCMC) [28, 29, 30, 31]. We used (random) ABC rejection sampling to initialise the MCMC chains. Where random sampling failed to find an adequate parameter set to initiate an MCMC chain, we used box-constrained optimisation to minimise the sum of $\hat{o}$. MCMC chains were then seeded with these optimised parameter sets to fully characterise the compatible parameter space.

The sampler tolerance, ϵ, was set equal to n, representing the maximum Euclidean distance of the normalised model outputs at which the ABC-MCMC sampler would accept parameter samples. Each MCMC chain was run for 10 million iterations, thinning accepted samples at a ratio of 100:1. Of the resulting 100,000 samples we randomly subsampled 1000 parameter sets to generate final results.

Supplementary Results

D. Calibration and Baseline Projections

D.1. ABC-MCMC Sampler Performance

Broadly, we achieved good mixing of MCMC chains over all models calibrated to all updated methods. The posterior parameter space compatible with model fit to all calibration targets was most constrained in the M3 model, as evidenced in the posterior density plots in Figure G, leading to comparatively reduced mixing compared to M0–2. MCMC chains for the 100,000 retained final samples for each calibrated model are presented in figures C–F.

D.2. Posterior Distributions

The posterior distributions for all parameters across calibrated models for M0–M3 are presented in Figure G. We found that the posterior parameter space compatible with full fit to calibration targets was consistently reduced in the M3 model compared to M0–M2.

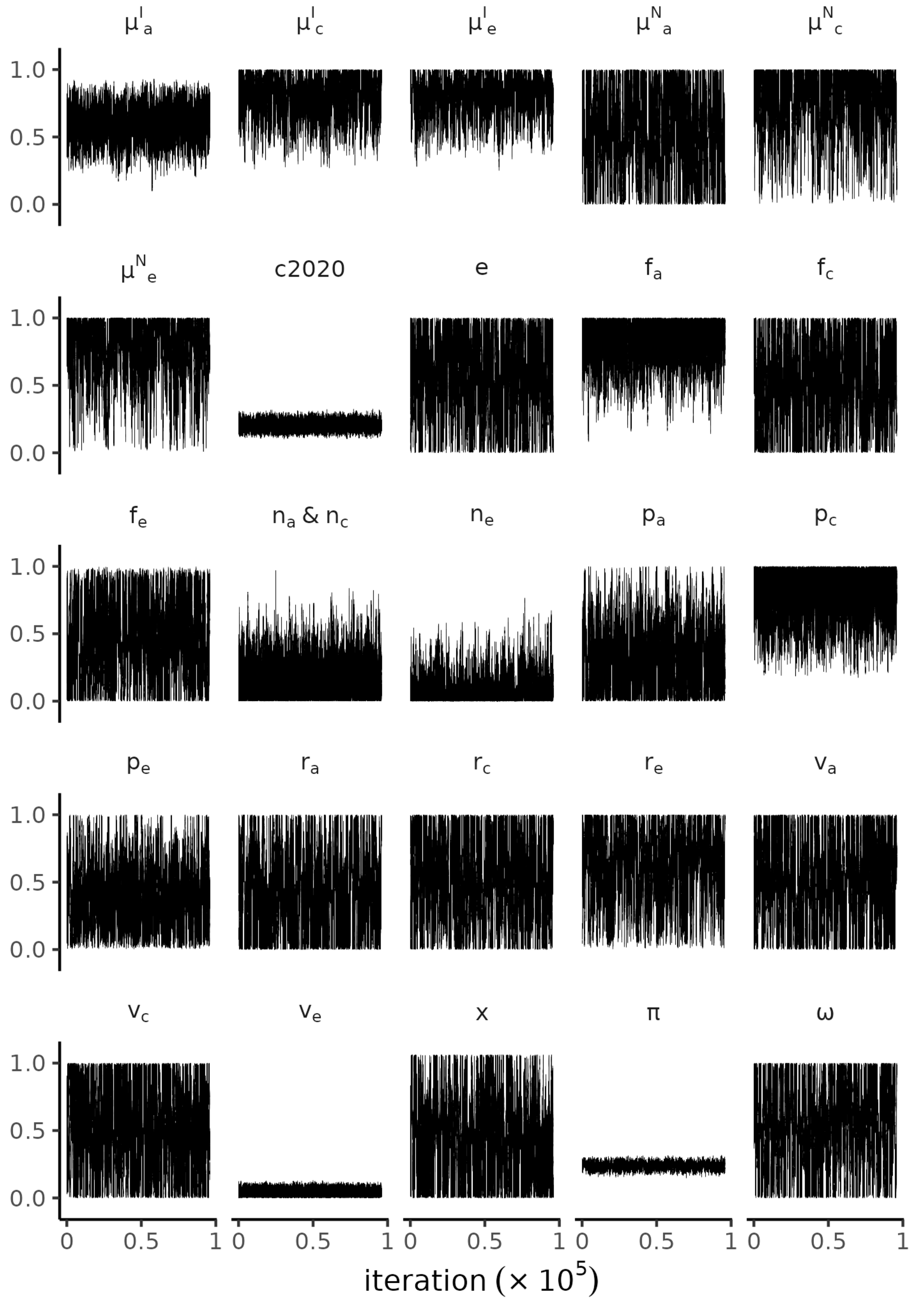

Figure C: MCMC chains generated during calibration of M0 model. 100,000 samples were retained per chain. Y-axes display parameter values normalised against their respective prior ranges.

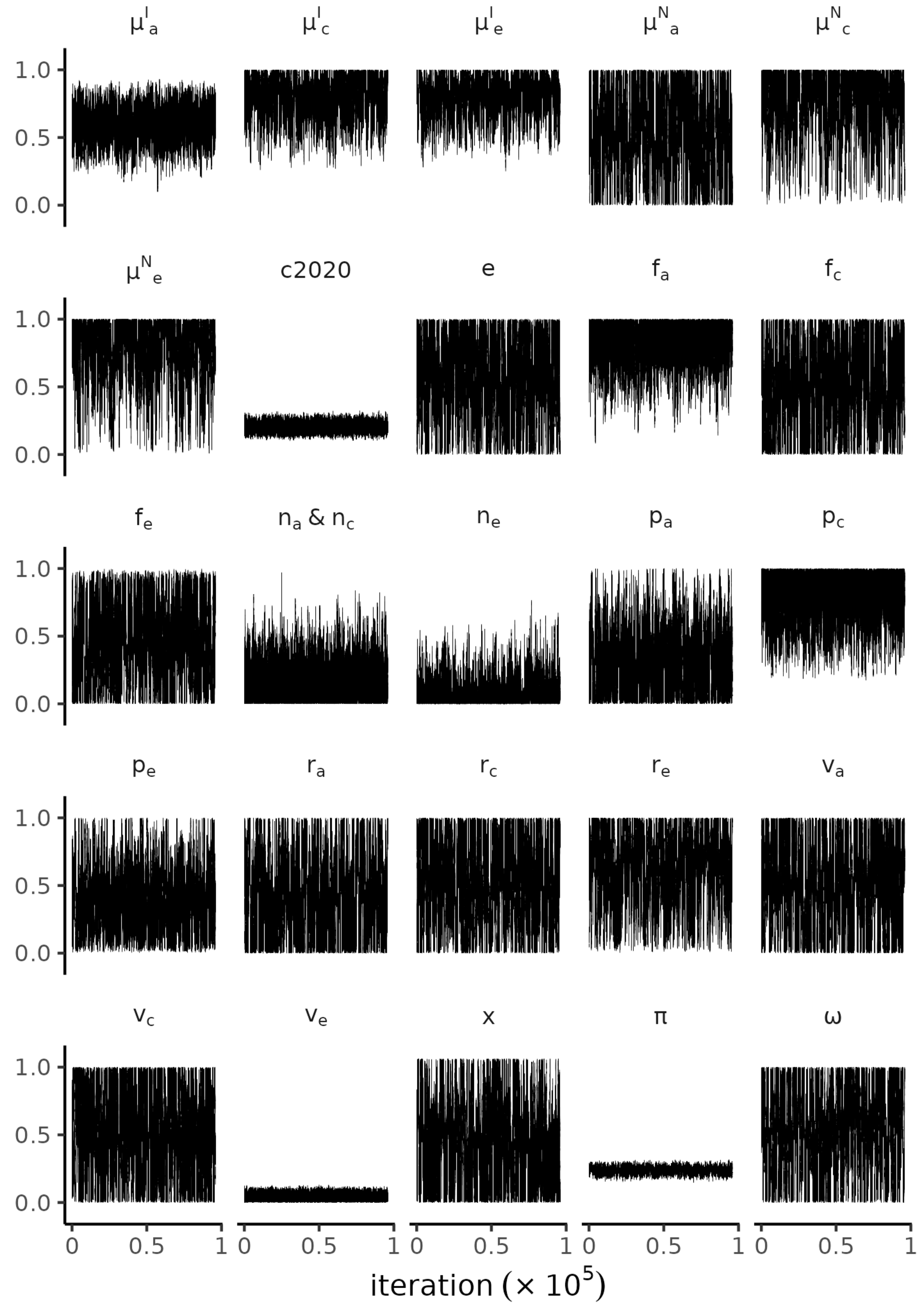

Figure D: MCMC chains generated during calibration of M1 model. 100,000 samples were retained per chain. Y-axes display parameter values normalised against their respective prior ranges.

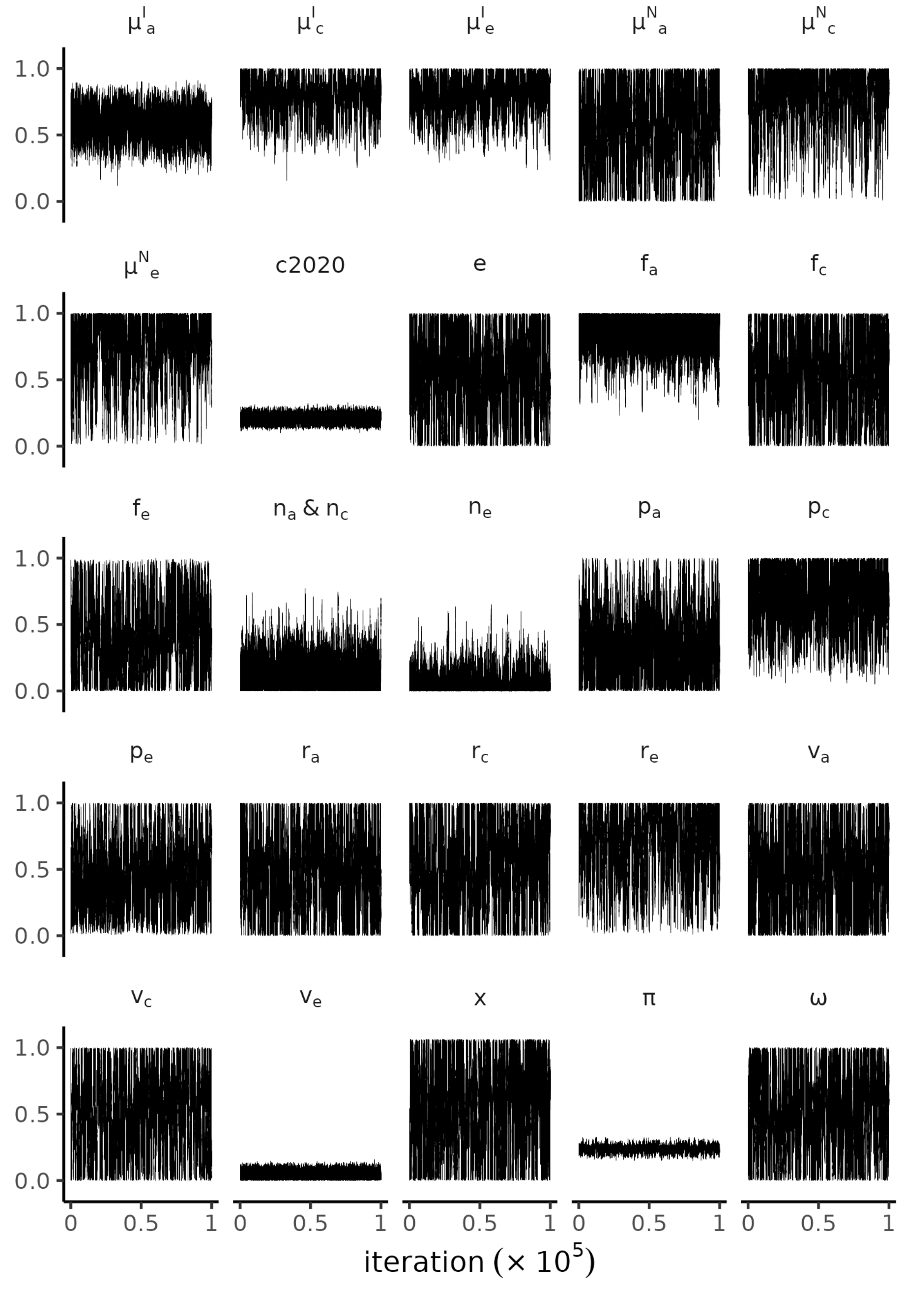

Figure E: MCMC chains generated during calibration of M2 model. 100,000 samples were retained per chain. Y-axes display parameter values normalised against their respective prior ranges.

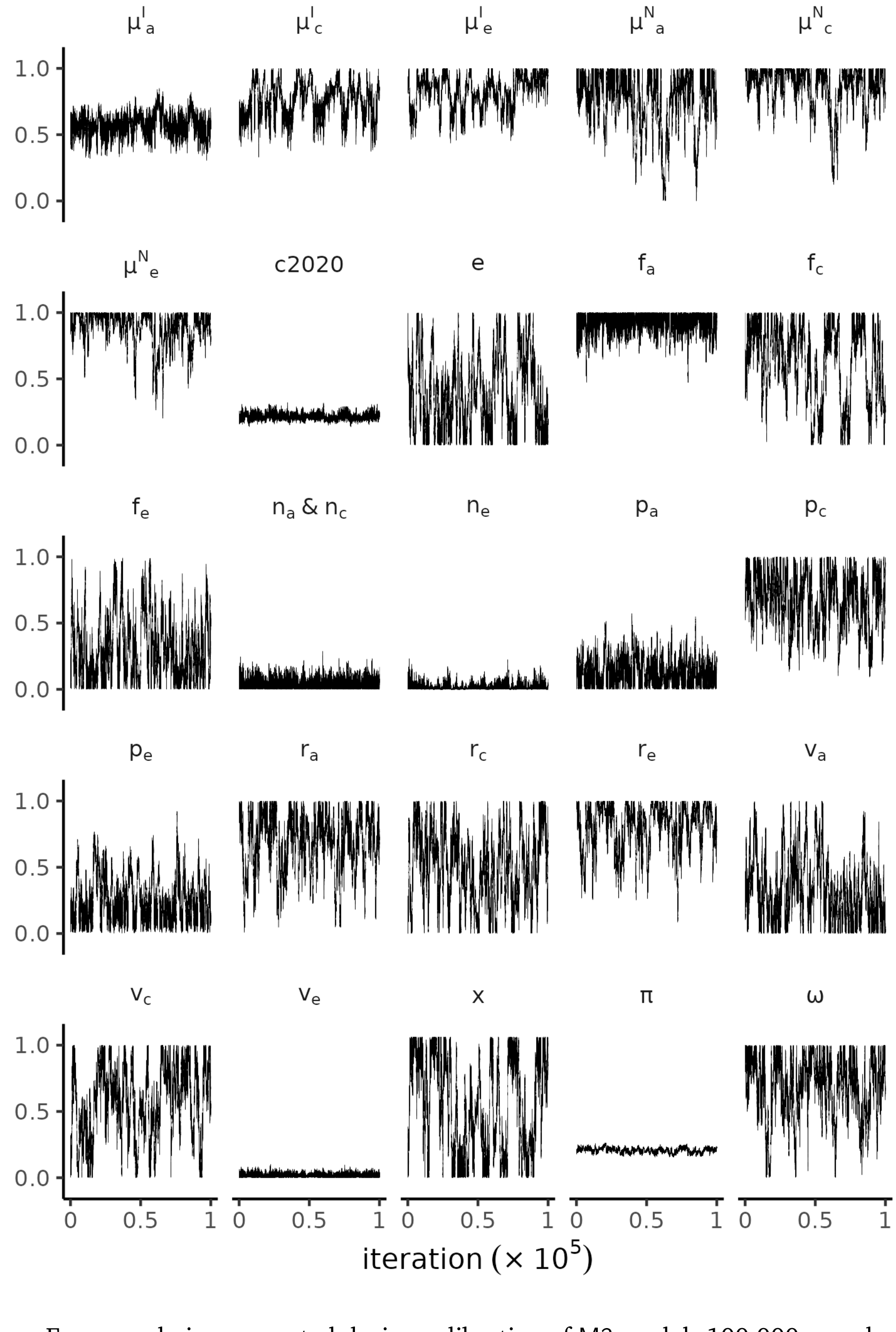

Figure F: MCMC chains generated during calibration of M3 model. 100,000 samples were retained per chain. Y-axes display parameter values normalised against their respective prior ranges.

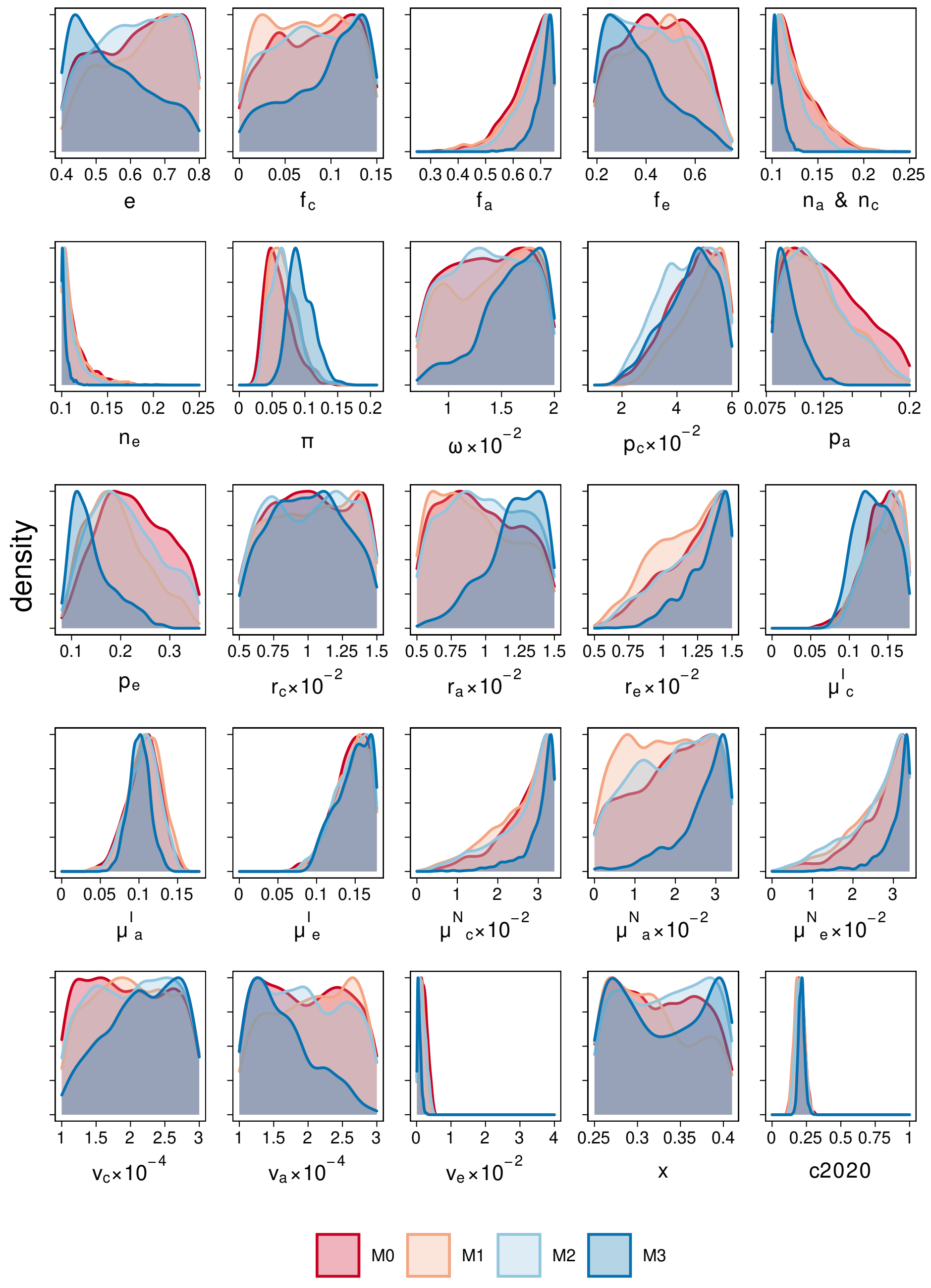

Figure G: Posterior distributions of model parameters. Y-axis values are normalised to a maximum value of one. X-axis limits represent the prior ranges of each parameter.

D.3. Baseline Projections

Calibration results for the M1 model and baseline incidence projections to 2050 are presented in figure H.

In the M1 model, prevalence rate was predicted to be 217 (uncertainty range: 195–312) per 100,000 in 2015, mortality rate was predicted to be 32 (UR: 30–35) per 100,000 and notification rate was predicted to be 167 (UR: 152–213) per 100,000 in 2019, respectively. Overall incidence rate was predicted to be 248 (UR: 206–293) per 100,000, 244 (UR: 205–265) per 100,000, and 234 (UR: 190–271) per 100,000 in 2010, 2019, and 2050 respectively.

As described in the main text, we applied the M0 2050 incidence rate value as a calibration target for the M0, M2, and M3 models.

Calibration results and baseline incidence projections to 2050 for the M0, M2, and M3 models are presented in Figures I, J and K respectively.

In the M0 model, prevalence rate was predicted to be 217 (UR: 195–310) per 100,000 in 2015, mortality rate was predicted to be 32 (UR: 30–35) per 100,000 and notification rate was predicted to be 168 (UR: 152–217) per 100,000 in 2019, respectively. Overall incidence rate was predicted to be 249 (UR: 205–288) per 100,000, 244 (UR: 206–265) per 100,000, and 239 (UR: 195–271) per 100,000 in 2010, 2019, and 2050 respectively.

In the M2 model, prevalence rate was predicted to be 213 (UR: 195–312) per 100,000 in 2015, mortality rate was predicted to be 32 (UR: 30–35) per 100,000 and notification rate was predicted to be 167 (UR: 152–221) per 100,000 in 2019, respectively. Overall incidence rate was predicted to be 234 (UR: 195–275) per 100,000, 242 (UR: 209–266) per 100,000, and 258 (UR: 213–271) per 100,000 in 2010, 2019, and 2050 respectively.

In the M3 model, prevalence rate was predicted to be 204 (UR: 195–273) per 100,000 in 2015, mortality rate was predicted to be 31 (UR: 30–35) per 100,000 and notification rate was predicted to be 161 (UR: 152–214) per 100,000 in 2019, respectively. Overall incidence rate was predicted to be 210 (UR: 180–263) per 100,000, 225 (UR: 206–263) per 100,000, and 266 (UR: 237–271) per 100,000 in 2010, 2019, and 2050 respectively.

D.3.1. Model Demography

Demographic projections for India from World Population Prospects for 2025–2050 are shown in figure L:**A** for the population overall and for the three age-groups of interest. Assuming υ_{U} as the World Population Prospected projected population value, and υ_{M} as the population estimate predicted by the M0–M3 models, we defined the relative error as:

$$\text{relative error} = \log_{10}\left(\frac{\upsilon_{\mathrm{U}} - \upsilon_{\mathrm{M}}}{\upsilon_{\mathrm{U}}}\right).$$

We found low relative error across all models, suggesting good concordance between projected demography and the underlying UN data (figure L:**B–E**). Error was smallest in the M3 model, likely reflecting the smaller fitted parameter space and ensuing reduced variability in model projections.

M1

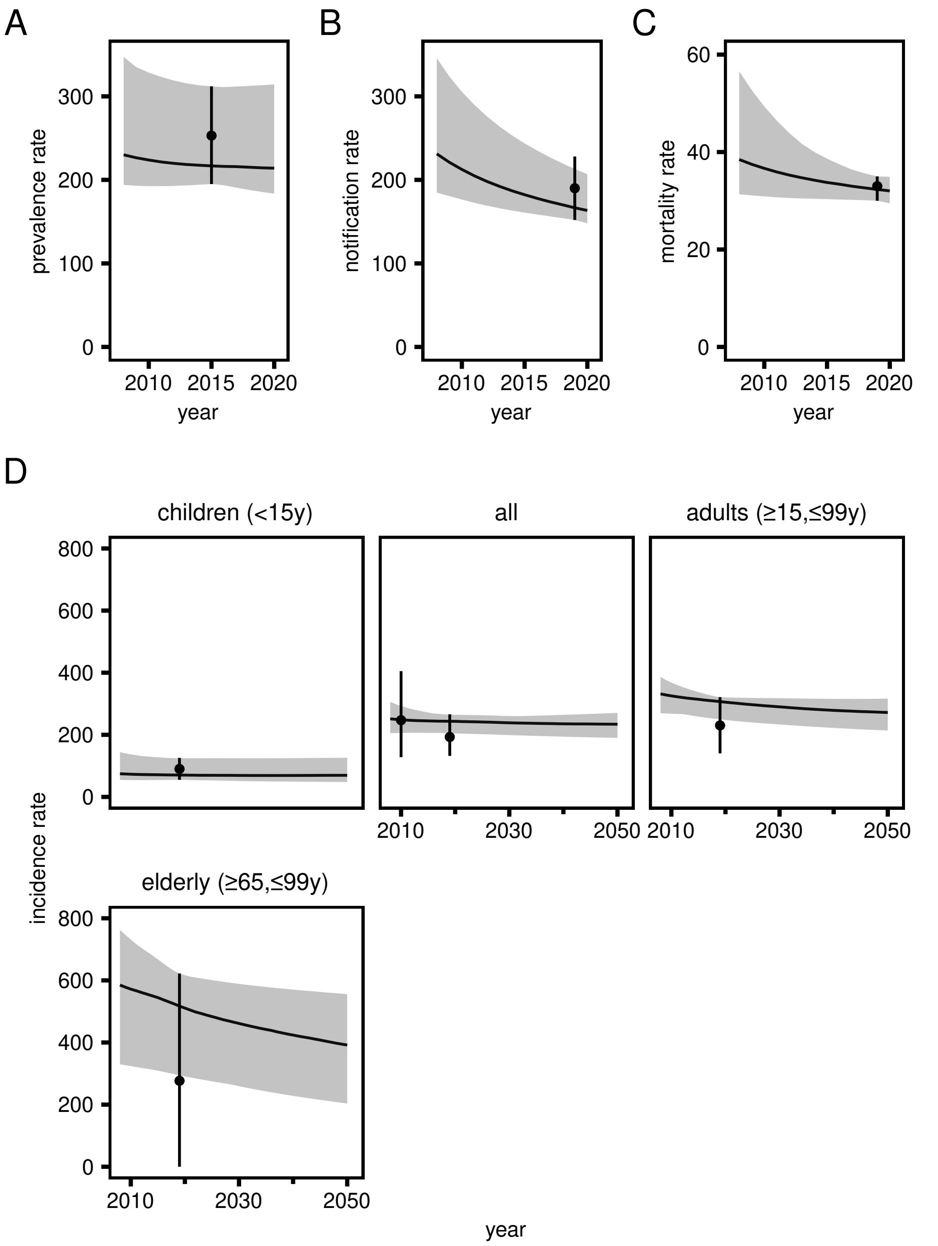

Figure H: Calibration and Baseline Projections—M1. Rates are specified per 100,000 population. Lines represent median estimates, ribbons represent uncertainty range. Points and bars represent calibration target and range, respectively. **A:** overall prevalence rate. **B:** overall notification rate. **C:** overall mortality rate. **D:** incidence rate, overall and disaggregated by age group, projected to 2050.

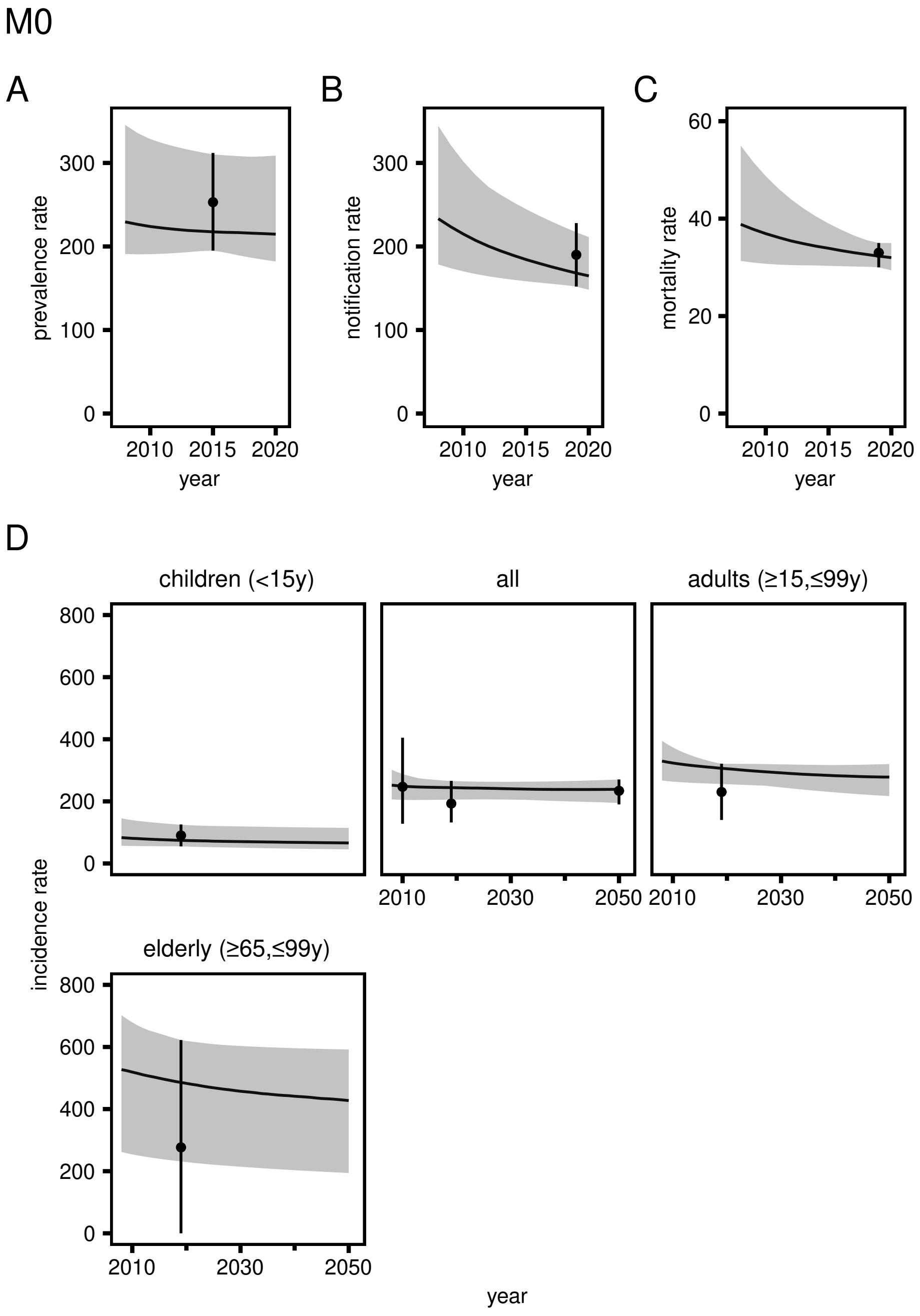

Figure I: Calibration and Baseline Projections—M0. Rates are specified per 100,000 population. Lines represent median estimates, ribbons represent uncertainty range. Points and bars represent calibration target and range, respectively. **A:** overall prevalence rate. **B:** overall notification rate. **C:** overall mortality rate. **D:** incidence rate, overall and disaggregated by age group, projected to 2050, including calibration target derived from model M1.

M2

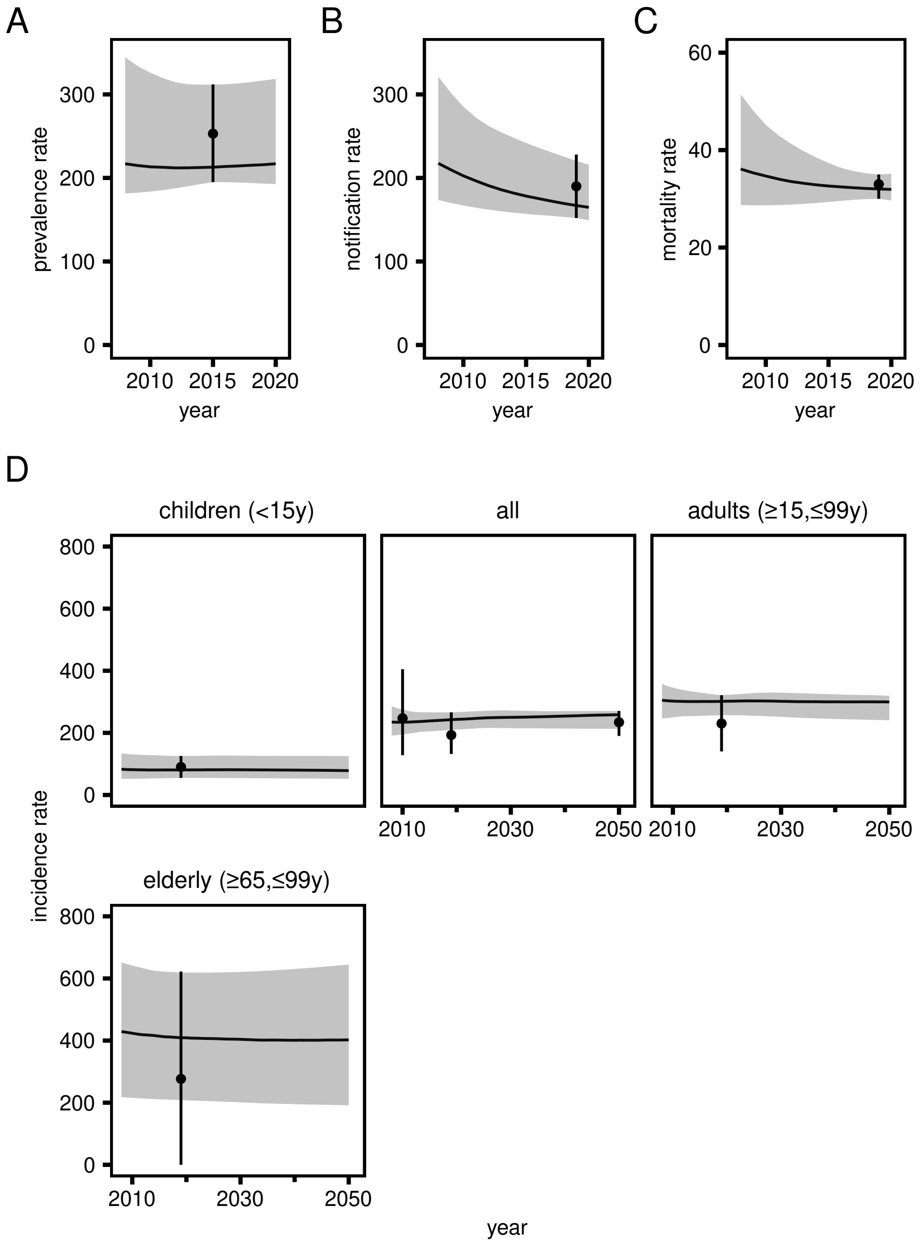

Figure J: Calibration and Baseline Projections—M2. Rates are specified per 100,000 population. Lines represent median estimates, ribbons represent uncertainty range. Points and bars represent calibration target and range, respectively. **A:** overall prevalence rate. **B:** overall notification rate. **C:** overall mortality rate. **D:** incidence rate, overall and disaggregated by age group, projected to 2050, including calibration target derived from model M1.

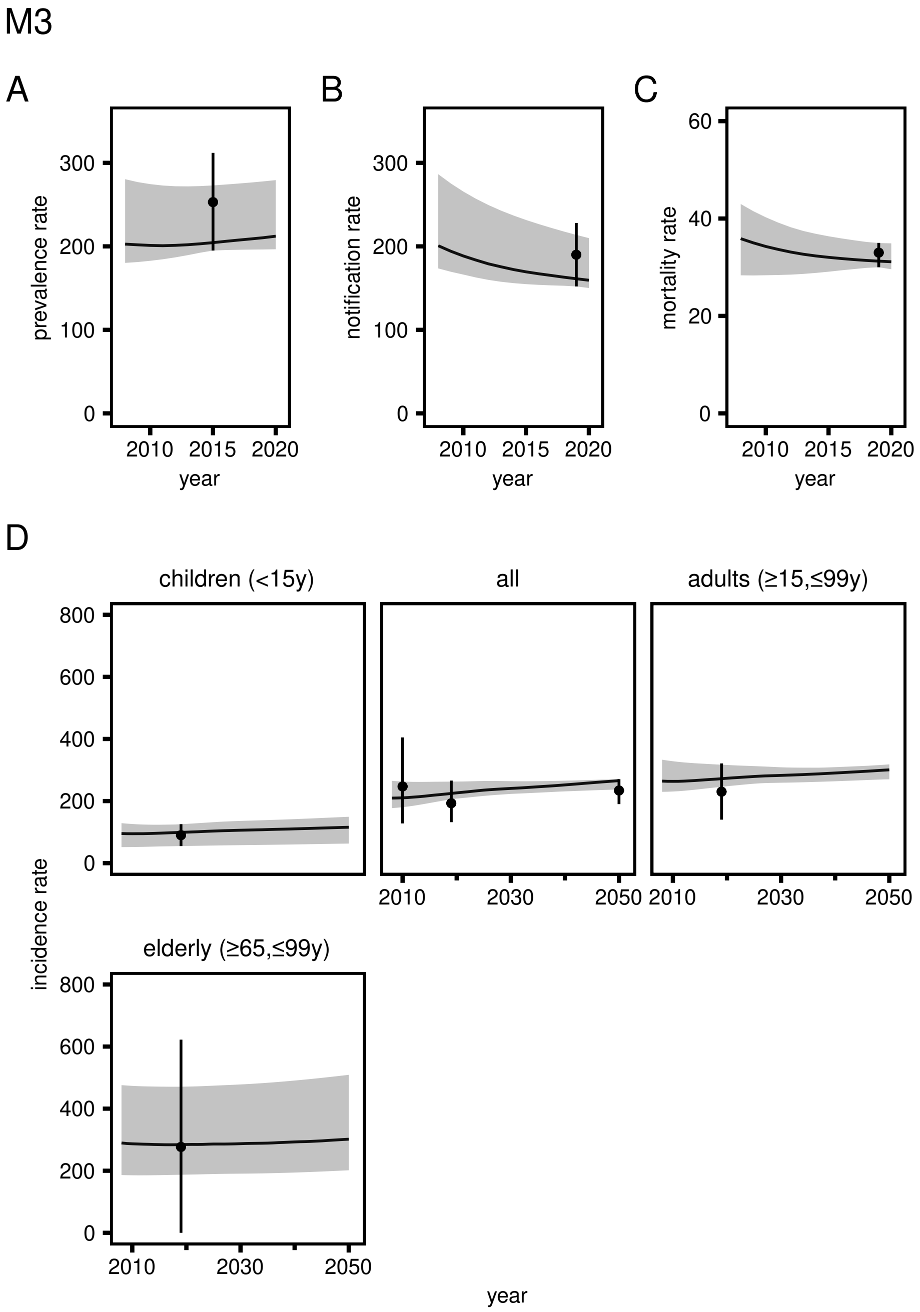

Figure K: Calibration and Baseline Projections—M3. Rates are specified per 100,000 population. Lines represent median estimates, ribbons represent uncertainty range. Points and bars represent calibration target and range, respectively. **A:** overall prevalence rate. **B:** overall notification rate. **C:** overall mortality rate. **D:** incidence rate, overall and disaggregated by age group, projected to 2050, including calibration target derived from model M1.

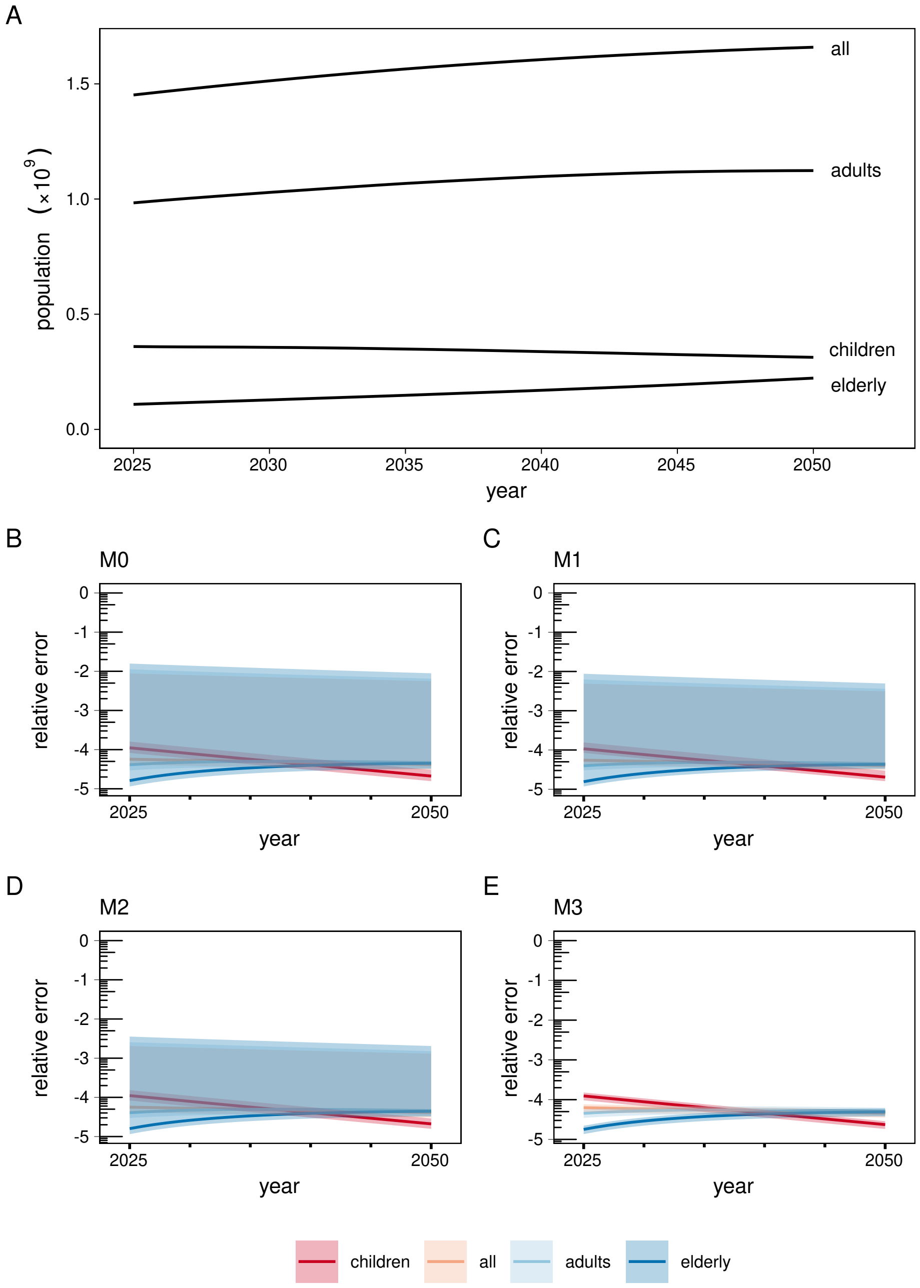

Figure L: Model demography. **A:** UN World Population Prospects (WPP) [6] medium projection—India country profile. **B–E:** Model relative error in update methods M0–M3. More negative relative error values indicate a lower discrepancy between the model projected demography and WPP demography.

D.3.2. Epidemic Analysis

Figure M shows TB incidence disaggregated by source. We find that the epidemic structure is broadly similar across M0–M3 models, largely driven by new infection followed by fast progression in naive individuals and recovered individuals, with a comparatively minor contribution from either reactivation or reinfection of latently infected individuals.

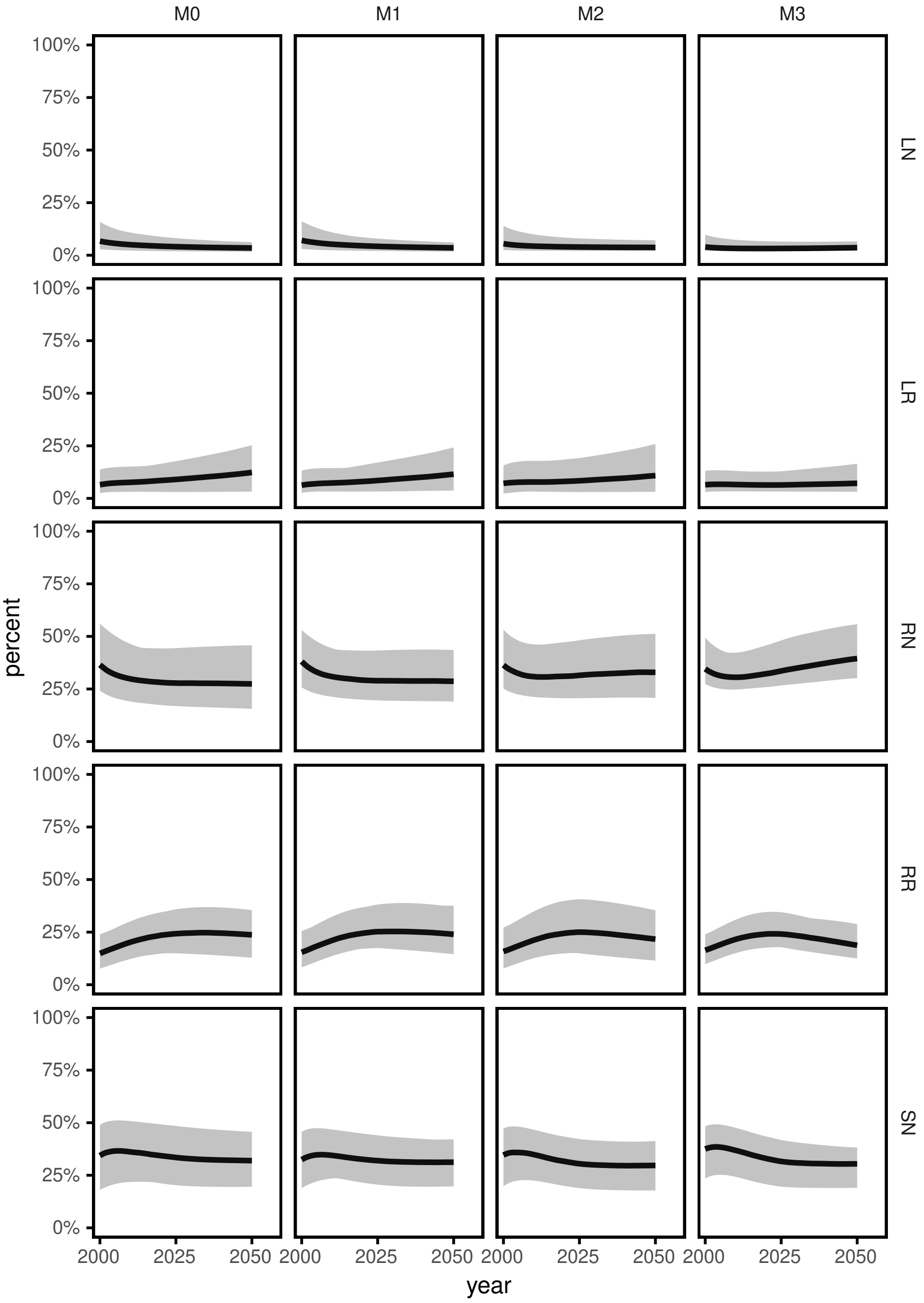

Figure M: Proportion of incident TB due to each transition-type. Rows represent the proportion of incident TB due to new infection and fastprogression of latently infected individuals (LN); reactivation in latent individuals (LR); new infection and fastprogression in recovered individuals (RN); relapse in recovered individuals (RR); and new infection followed by fastprogression in susceptible individuals (SN). Columns represent M0–M3 updated models. Lines represent median estimates, ribbons represent uncertainty ranges.

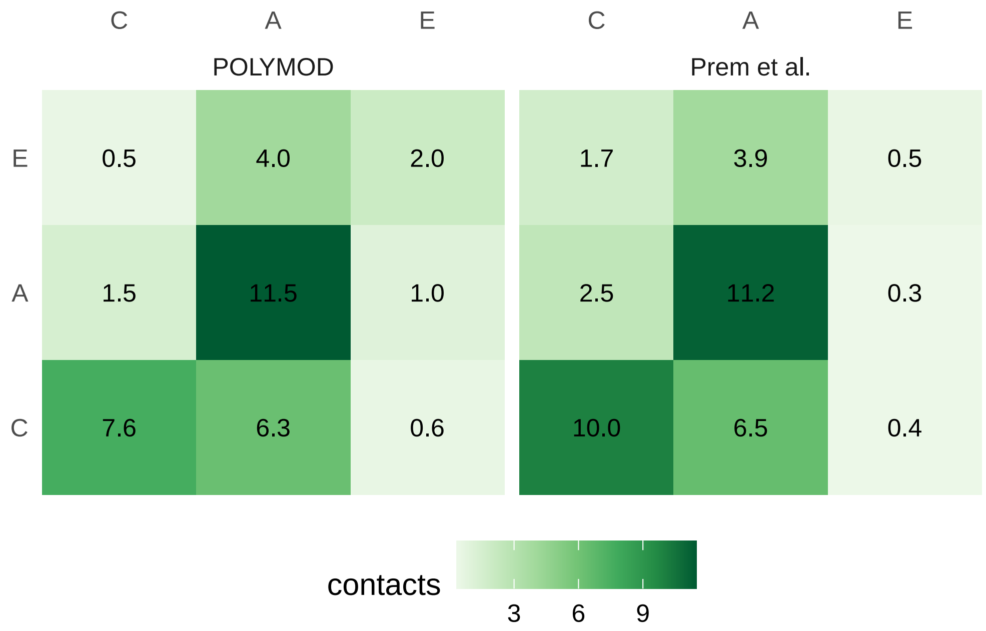

Figure N: Comparison: Prem et al vs unadjusted POLYMOD. C=children; A=adults; E=elderly. Matrices show contacts per day.

E. Vaccine Impact

Vaccine impact across models M0–M3 is summarised in table C, including a sensitivity analysis for host infection status required for efficacy. We find that differential vaccine impact across update methods remains insensitive to host-infection status required for efficacy. Similar to PSI vaccines, the maximum difference in impact in the elderly following adulttargeted vaccination at 8% between M0 and M3 scenarios.

F. Country Adjusted Matrix

Figure N shows the unadjusted base POLYMOD contact matrix used in this study, in comparison to that derived from Prem et al [32]. We find that both contact rate magnitudes and assortativity patterns remain broadly similar.

Table C: Percent TB incidence rate reduction due to vaccine compared to no-vaccine in 2050, over four contact matrix update scenarios. The vaccine is assumed to have been delivered at 70% coverage and 10-yearly mass campaigns beginning in 2027. Values represent median (uncertainty interval).

Host Status	Target Group	Update Method	Outcome Group		
			children	adults	elderly
P&PI	children	M0	33% (30–35)	5% (4–7)	2% (1–4)
		M1	33% (30–35)	5% (4–8)	2% (1–4)
		M2	33% (30–36)	6% (4–8)	2% (1–5)
		M3	33% (30–35)	6% (5–8)	3% (2–5)
	adults	M0	43% (34–53)	55% (49–63)	23% (15–38)
		M1	45% (35–55)	55% (50–63)	25% (15–38)
		M2	46% (36–57)	58% (51–66)	27% (16–43)
		M3	47% (39–56)	59% (55–67)	31% (20–45)
	elderly	M0	4% (1–9)	3% (1–7)	31% (29–36)
		M1	3% (1–7)	2% (1–6)	31% (29–35)
		M2	2% (1–6)	2% (1–5)	30% (28–35)
		M3	1% (1–3)	1% (0–3)	29% (28–32)
PRI	children	M0	24% (17–27)	4% (3–6)	1% (1–3)
		M1	24% (19–28)	4% (3–6)	2% (1–3)
		M2	23% (17–27)	4% (3–7)	2% (1–4)
		M3	22% (17–26)	5% (4–6)	2% (1–4)
	adults	M0	12% (5–23)	17% (8–28)	6% (2–14)
		M1	12% (5–25)	17% (9–32)	6% (2–16)
		M2	12% (4–24)	16% (7–29)	7% (2–16)
		M3	9% (5–19)	13% (8–25)	6% (3–15)
	elderly	M0	0% (0–1)	0% (0–1)	3% (1–8)
		M1	0% (0–1)	0% (0–1)	3% (1–8)
		M2	0% (0–1)	0% (0–1)	3% (1–7)
		M3	0% (0–0)	0% (0–0)	2% (1–6)
PSI	children	M0	10% (7–16)	1% (1–2)	0% (0–1)
		M1	10% (6–15)	1% (1–2)	0% (0–1)
		M2	11% (7–17)	1% (1–3)	1% (0–1)
		M3	12% (9–16)	2% (1–3)	1% (1–2)
	adults	M0	34% (28–41)	44% (38–50)	19% (13–32)
		M1	35% (29–42)	44% (38–51)	20% (13–31)
		M2	37% (29–44)	47% (41–55)	22% (14–37)
		M3	39% (32–48)	50% (44–59)	26% (18–38)
	elderly	M0	4% (1–8)	3% (1–7)	28% (24–33)
		M1	3% (1–7)	2% (1–5)	28% (24–31)
		M2	2% (1–5)	2% (1–4)	27% (23–32)
		M3	1% (0–3)	1% (0–3)	27% (24–29)

References

1. Knight, G. M. *et al.* Impact and Cost-Effectiveness of New Tuberculosis Vaccines in Low- and Middle-Income Countries. *Proc Natl Acad Sci U S A* **111,** 15520–15525. ISSN: 0027-8424, 1091-6490 (Oct. 28, 2014).
2. Harris, R. C. *et al.* Age-Targeted Tuberculosis Vaccination in China and Implications for Vaccine Development: A Modelling Study. *Lancet Glob Health* **7,** e209–e218. ISSN: 2214-109X (Feb. 1, 2019).
3. Harris, R. C., Sumner, T., Knight, G. M., Zhang, H. & White, R. G. Potential Impact of Tuberculosis Vaccines in China, South Africa, and India. *Sci Transl Med* **12.** ISSN: 1946-6234, 1946-6242 (Oct. 7, 2020).
4. Weerasuriya, C. K. *et al.* The Epidemiologic Impact and Cost-Effectiveness of New Tuberculosis Vaccines on Multidrug-Resistant Tuberculosis in India and China. *BMC Med* **19,** 60. ISSN: 1741-7015 (Feb. 26, 2021).
5. Weerasuriya, C. K. *et al.* Affordability of Adult Tuberculosis Vaccination in India and China: A Dynamic Transmission Model-Based Analysis. *Vaccines (Basel)* **9,** 245 (3 Mar. 2021).
6. United Nations Department of Economic and Social Affairs, Population Division. *World Population Prospects 2019* 2 vols. ISBN: 978-92-1-148328-4 (2019).
7. Abu-Raddad, L. J. *et al.* Epidemiological Benefits of More-Effective Tuberculosis Vaccines, Drugs, and Diagnostics. *Proc Natl Acad Sci U S A* **106,** 13980–13985. ISSN: 0027-8424, 1091-6490 (Aug. 2009).
8. Dye, C., Garnett, G. P., Sleeman, K. & Williams, B. G. Prospects for Worldwide Tuberculosis Control under the WHO DOTS Strategy. *Lancet* **352,** 1886–1891. ISSN: 0140-6736, 1474-547X (Dec. 12, 1998).
9. Marx, F. M. *et al.* The Temporal Dynamics of Relapse and Reinfection Tuberculosis After Successful Treatment: A Retrospective Cohort Study. *Clin Infect Dis* **58,** 1676–1683. ISSN: 1058-4838 (June 15, 2014).
10. Keeling, M. J. & Rohani, P. *Modeling Infectious Diseases in Humans and Animals* 366 pp. ISBN: 978-0-691-11617-4 (Princeton University Press, Princeton, 2008).
11. Arregui, S., Aleta, A., Sanz, J. & Moreno, Y. Projecting Social Contact Matrices to Different Demographic Structures. *PLoS Comput Biol* **14,** e1006638. ISSN: 1553-7358 (Dec. 7, 2018).
12. Mossong, J. *et al.* Social Contacts and Mixing Patterns Relevant to the Spread of Infectious Diseases. *PLoS Med* **5,** e74. ISSN: 1549-1676 (Mar. 25, 2008).
13. Newman, M. E. J. Mixing Patterns in Networks. *Phys. Rev. E* **67,** 026126. ISSN: 1063-651X, 1095-3787 (Feb. 27, 2003).
14. World Health Organization. *WHO Tuberculosis Database* `http://www.who.int/tb/country/data/download/en/` (2021).
15. Dye, C. & Williams, B. G. Eliminating Human Tuberculosis in the Twenty-First Century. *J R Soc Interface* **5,** 653–662. ISSN: 1742-5689, 1742-5662 (2008).

16. Vynnycky, E. & Fine, P. E. The Natural History of Tuberculosis: The Implications of Age-Dependent Risks of Disease and the Role of Reinfection. *Epidemiol Infect* **119,** 183–201. ISSN: 0950-2688. `https://www.ncbi.nlm.nih.gov/pmc/articles/PMC2808840/` (2017) (Oct. 1997).
17. Gomes, G. M. *et al.* Implications of Partial Immunity on the Prospects for Tuberculosis Control by Post-Exposure Interventions. *J Theor Biol* **248,** 608–617. ISSN: 0022-5193 (Oct. 2007).
18. Schaaf, H. S., Collins, A., Bekker, A. & Davies, P. D. O. Tuberculosis at Extremes of Age. *Respirology* **15,** 747–763. ISSN: 1323-7799, 1440-1843 (July 2010).
19. Schulzer, M., Fitzgerald, J. M., Enarson, D. A. & Grzybowski, S. An Estimate of the Future Size of the Tuberculosis Problem in Sub-Saharan Africa Resulting from HIV Infection. *Tuber Lung Dis* **73,** 52–58. ISSN: 0962-8479 (Feb. 1992).
20. Rajagopalan, S. & Yoshikawa, T. T. Tuberculosis in the Elderly. *Z Gerontol Geriatr* **33,** 374–380. ISSN: 0948-6704 (Oct. 2000).
21. Yoshikawa, T. T. Tuberculosis in Aging Adults. *J Am Geriatr Soc* **40,** 178–187. ISSN: 0002-8614 (Feb. 1992).
22. High, K. & Marion, C. in *Infectious Disease in the Aging: A Clinical Handbook* (eds Yoshikawa, T. & Norman, D.) 2nd ed., 97–110 (Humana Press, Totowa, New Jersey, USA, June 12, 2009). ISBN: 978-1-60327-534-7.
23. Ferebee, S. H. Controlled Chemoprophylaxis Trials in Tuberculosis. A General Review. *Bibl Tuberc* **26,** 28–106. ISSN: 0300-1121 (1970).
24. Dye, C. Making Wider Use of the World's Most Widely Used Vaccine: Bacille Calmette-Guerin Revaccination Reconsidered. *J R Soc Interface* **10,** 20130365. ISSN: 1742-5662 (Oct. 6, 2013).
25. Tiemersma, E. W., Werf, M. J. van der, Borgdorff, M. W., Williams, B. G. & Nagelkerke, N. J. D. Natural History of Tuberculosis: Duration and Fatality of Untreated Pulmonary Tuberculosis in HIV Negative Patients: A Systematic Review. *PLoS One* **6,** e17601. ISSN: 1932-6203 (Apr. 4, 2011).
26. World Health Organization. *Global Tuberculosis Report 2019* ISBN: 978-92-4-156571-4 (World Health Organization, Geneva, Switzerland, 2019).
27. Pandey, S., Chadha, V. K., Laxminarayan, R. & Arinaminpathy, N. Estimating Tuberculosis Incidence from Primary Survey Data: A Mathematical Modeling Approach. *Int J Tuberc Lung Dis* **21,** 366–374. ISSN: 1027-3719 (Apr. 2017).
28. Toni, T., Welch, D., Strelkowa, N., Ipsen, A. & Stumpf, M. P. H. Approximate Bayesian Computation Scheme for Parameter Inference and Model Selection in Dynamical Systems. *J R Soc Interface* **6,** 187–202. ISSN: 1742-5689, 1742-5662 (Feb. 6, 2009).
29. Toni, T. & Stumpf, M. P. H. *Tutorial on ABC Rejection and ABC SMC for Parameter Estimation and Model Selection* Oct. 23, 2009. `http://arxiv.org/abs/0910.4472` (2018).

30. Wegmann, D., Leuenberger, C. & Excoffier, L. Efficient Approximate Bayesian Computation Coupled With Markov Chain Monte Carlo Without Likelihood. *Genetics* **182,** 1207–1218. ISSN: 0016-6731, 1943-2631 (Aug. 1, 2009).

31. Marjoram, P., Molitor, J., Plagnol, V. & Tavare, S. Markov Chain Monte Carlo without Likelihoods. *Proc Natl Acad Sci U S A* **100,** 15324–15328. ISSN: 0027-8424, 1091-6490 (Dec. 23, 2003).

32. Prem, K. *et al.* Projecting Contact Matrices in 177 Geographical Regions: An Update and Comparison with Empirical Data for the COVID-19 Era. *PLOS Computational Biology* **17,** e1009098. ISSN: 1553-7358 (July 26, 2021).
