# Supplementary material for: Updating age-specific contact structures to match evolving demography in a dynamic mathematical model of tuberculosis vaccination
Source: PLoS Comput Biol. 2022 Apr 22;18(4):e1010002. doi: 10.1371/journal.pcbi.1010002 (PMC9067655; doi:10.1371/journal.pcbi.1010002)
Supplement: Attachment — Submitted filename: PCOMPBIOL-D-21-01594.pdf [file pcbi.1010002.s002.pdf]

Review

Manuscript ID: PCOMPBIOL-D-21-01594
Paper Title: Updating contact structures to match evolving demography in a dynamic mathematical model of tuberculosis vaccination

The authors investigated the effects of updating age-specific contact matrices to match the changing demography on the estimates for vaccination impact. The subject of the paper is interesting and important, and the paper is overall well written.

1. I think it helps to clearly define upfront what a "contact matrix" refers to. Does it represent the average (daily) number of contacts that an individual in age group i makes with an individual in age group j? Does it represent the (daily) number of contacts between the members of age groups i and j (in which case, the matrix should be symmetrical).
2. Page 8, lines 154-156: Would you please provide the details of how the contact matrices of Fig 1A are generated from the raw POLYMOD contact data?
3. Page 10, lines 195-204: It is not clear why β (the scaled per-capita effective contact rate) is introduced. It might make sense to move the mathematical definition of β from the supplement to the main text. Also, in page 6 of the supplement, β seems to be representing both a matrix and a scalar.

Minor issues:

4. Page 6 of the supplement: at the last paragraph, the references to figures missing.
5. Page 7 of the supplement: It is not clear what "contact matrix2.2" is referring to.
