# Supplementary material for: Updating age-specific contact structures to match evolving demography in a dynamic mathematical model of tuberculosis vaccination
Source: PLoS Comput Biol. 2022 Apr 22;18(4):e1010002. doi: 10.1371/journal.pcbi.1010002 (PMC9067655; doi:10.1371/journal.pcbi.1010002)
Supplement: Attachment — Submitted filename: PCOMPBIOL-D-21-01594_Response to reviewers.pdf [file pcbi.1010002.s003.pdf]

TB Modelling Group and TB Centre,
Department for Infectious Disease Epidemiology
London School of Hygiene & Tropical Medicine
Keppel Street, London WC1E 7HT
+44 (0)20 7636 8636 • www.lshtm.ac.uk

Jan 26, 2022

Re: PCOMPBIOL-D-21-01594 — Updating Contact Structures to Match Evolving Demography in a Dynamic Mathematical Model of Tuberculosis Vaccination

Dear Editors,

Thank you for the high quality and comprehensive review of our paper and for your helpful and constructive comments. We appreciate your, and the reviewers', time spent critically appraising our manuscript to highlight important issues that can be addressed.

A response to each reviewer comment is given in the table below. We have altered the manuscript where relevant and indicated the changes within it using the "Track Changes" feature. The edits to the manuscript text are cross-referenced to manuscript line numbers in the table below.

In addition, we have made some changes to the text in the Introduction section to improve readability. These are indicated through tracked changes in the resubmitted manuscript.

We hope that these revisions fully address the issues raised by the reviewers and look forward to your correspondence.

Chathika Weerasuriya
On behalf of all authors

<table>
<tr><th>Review comments</th><th>Response</th></tr>
<tr><td>Reviewer 1</td><td></td></tr>
<tr><td>This manuscript presents a well written if rather technical modelling analysis of the impact of assumptions of contact structures on results of a mathematical transmission model of TB in India. In essence, the authors' work shows that they don't, so it is a "negative" result.

I am not an expert in TB modelling, so I cannot assess the relevance and appropriateness of the modelling assumptions, although they appear to make sense.

MAJOR COMMENTS

One major issue is that their assessment relies quite heavily on a contact structure from the POLYMOD paper, which is based on data collected from Europe. It would be important to conduct some form of sensitivity analysis, e.g. using possibly data from a different setting, which might be a better reflection of social interactions in India.</td><td>Please note all line numbers are with “No Markup” enabled in the Track Changes mode.

We thank the reviewer for highlighting this important issue. In summary, we used POLYMOD data due to three factors:

(i) POLYMOD contains large, high quality, nationally representative data, whereas the currently available India-specific or India-like empirical data were from smaller, non-nationally representative, communities and not published in a form we could use in our study; (ii) the overall aggregate assortativity patterns of these smaller surveys or synthetic estimates were very similar to the POLYMOD data; and (iii) post hoc adjustment (following reviewer feedback) of the POLYMOD data for household, classroom, and workplace structures in India and into the age-groups used in our model led to a very similar matrix to an unadjusted POLYMOD matrix.

Especially due to factors (ii) and (iii), it is unlikely that using an alternative base contact matrix would alter our conclusions.

We give more details supporting our summary below.

1. To date, there are no nationally representative empirically derived contact data for India. Existing surveys are from smaller, non-nationally representative, communities (e.g., Kumar et al. [1]) or synthetic (Prem et al. [2]).
Raw data of a form suitable to construct matrices for our model are not published for subnational surveys from other settings, e.g., by Kumar et al. (India) [1] or Kiti et al. (Kenya) [3]. The contact estimates published by Read et al. (China) [4] focus on differential contact patterns by level of urbanisation and show a broadly similar pattern in total contact rate and duration by age, albeit of a slightly greater magnitude, to POLYMOD. Our work does not stratify by location or urbanisation, so we could not incorporate this heterogeneity. Nonetheless, this may serve as an important avenue warranting further work, so we have added this point to the manuscript</td></tr>
</table>

	**discussion (below; lines 414–425).**<br><br>2. The assortativity patterns seen in these smaller/synthetic surveys were similar to the POLYMOD matrix we used, particularly at our level of age-group aggregation. **Points (1) and (2) are specifically addressed in the manuscript discussion as follows (lines 414–425):**<br>*Additionally, our use of a generic POLYMOD contact matrix rather than an India-specific matrix may reduce the accuracy of our findings. We know of two previous studies which estimated social contact patterns in India. Prem et al. [3] combined Demographic and Health Survey results, POLYMOD data, and other household-level data to generate synthetic age-specific contact matrices for India. Kumar et al. [7] reported a social contact survey limited to Haryana, North India. At the time of writing, raw data from neither study was available in the form needed to generate matrices for our model. However, similarly to the POLYMOD matrix, both studies found strong assortativity for in-age-group contacts, with additional assortativity between younger adults and children. Therefore, we speculate that differential impact across update methods is likely to remain stable, despite possible different magnitudes of vaccine impact and age-specific impact patterns.*<br><br>3. In response to reviewer feedback, post hoc, we analysed whether the POLYMOD contact matrix, used at our level of age-group aggregation, was likely biased by differences in household structure, classroom, and workplace composition between India and the POLYMOD countries. In July 2021, after the completion of our study, Prem et al. published updated estimates of synthetic contact matrices for 177 countries [5], including India. These matrices were derived by adjusting the POLYMOD data with additional country-level data on sociodemographics, household structure, employment and school attendance (from the UN World Population Prospects, DHS and other sources). We used the total raw contacts predicted for India by Prem et al. to generate a three-age class matrix equivalent to our study. The resulting matrix (included in the technical appendix §6) was very similar to the (unadapted) POLYMOD matrix used in our study. This analysis suggests that while the household structure in India will likely differ from POLYMOD countries, at nationally aggregated levels with the age groups used in our study, these differences are unlikely to bias our results substantially.<br><br>**We have now specifically included this analysis in the manuscript as follows:**<br>a. **Methods Lines 235–244:** *Country Adapted Matrix*<br>*In July 2021, Prem et al [21] published an updated set of synthetic contact matrices for 177 countries derived by adapting POLYMOD data to reflect country-level demo-graphic, household, and location-specific composition (home, work, school, other) characteristics using UN World Population Prospects demographic*

projections, Demo-graphic and Health Survey data, and other sources. We used the estimated number of total contacts predicted for India by Prem et al to generate an equivalent matrix to Fig 1A. We compared this back-calculated matrix to that in Fig 1A to assess if country-specific adjustments led to substantially different contact rates or assortativity at this level of age-group aggregation.

b. **Results Lines 334–336:** *Finally, we found that adjustment for India-specific sociodemographic features did not lead to a substantially different base contact matrix compared to Fig 1A (appendix §6) at this level of aggregation of age-groups.*
c. **Discussion Lines 425–430**: *Furthermore, we found that adjustment for different household structure, classroom, and workplace composition did not substantially change the assortativity patterns of our base contact matrix. This analysis suggests that while household structure in India will likely differ from POLYMOD countries, at nationally aggregated levels with the age groups used in our study, these differences are unlikely to substantially bias our results.*

Finally it was also not clear to me whether the level of future vaccination coverage could play a role for results.

In previous work, using similarly structured models also set in India [6–8], we have consistently found that vaccine impact behaved relatively linearly with coverage. On this basis, we presume that relative differences in vaccine impact between different contact matrix update methods are likely to be preserved over changes in immunisation coverage. **We have added this explanation in the technical appendix section 2.5**.

It would also be important to make the model code available to other researchers in a public depostory.

Model code has been uploaded to GitHub and a snapshot is provided through Zenodo: https://doi.org/10.5281/zenodo.5902656
We have indicated this in the section "Code" at the end of the manuscript (lines 487–489)

MINOR COMMENTS
p. 3 l.45 Vaccines can hardly be considered to be new tools. Please rephrase.
p. 23 Fig. 1 Describe what C, A & E means in legend.
p. 27 Update Citation 8. This has in the mean time been published in PNAS

Appendix:
p. 6 penultimate line Update Figure crossref.
p. 9 Figure 2 C: Please detail which lines are overlaid.
p. 21 line 2. sentence needs to be completed.
p. 28 missing in fastprogression
p. 29 missing space in adulttargeted

Thank you for these comments; we have corrected these errors.

**Reviewer 2**	
The authors use and adapt a version of a previously developed and published model to explore whether temporal changes in age-specific contact patterns will have implications for the population-level impact of adult TB vaccines. The authors model four different ways in which age-specific contacts change over time, which is adapted from a previous study exploring how empirical data on age-specific contact can be applied in general. Specifically, the authors explore models that incrementally<br>1. forces "reciprocity" in the number of between-age-group contacts at the size of age-groups change;<br>2. preserves "assortativity" in between-age-group contacts or the propensity to contact someone in certain age-group over others; and<br>3. preserves the total number of contacts in the population as the population sizes change.<br>The methods are very well detailed in the supplementary materials, and the findings are described fairly well.<br>The authors find that in the context of the epidemiological impact of adult TB vaccines that is deployed in a high burden setting like India, these do not significantly change the results.<br><br>Overall, I find this study methodologically sound, and the questions addressed to be of significance (but perhaps not as well motivated).<br><br>My main suggestions for the authors are as follows:	**Please note all line numbers are with "No Markup" enabled in the Track Changes mode.**
1. The main question, why and how these specific features about translating empirical data on contact matrices matter, is not motivated adequately. This could partially be addressed by	We thank the reviewer for their very useful feedback. We have restructured the manuscript to better motivate why and how these specific features about translating empirical data on contact matrices matter, as follows:
i. describing in what form these empirical data are, and why precisely these modelling choices have to be made -- currently, it relies on the authors reading previous papers to understand these precisely;	(i) In lines 96–100 we draw the link between a particular contact survey and the demography of its source population. **We have added a description of the contact survey process (Introduction - lines 80–84) as follows:**<br>- *Most often, contact surveys request that participants record every contact they have with others within a fixed time period in a diary, where a contact is defined as at least a skin-to-skin (e.g., a handshake) or a two-way conversation. Participants are typically recruited to reflect the sociodemographic characteristics of their source populations.*
ii. laying out the potential reasonings behind a specific model (beyond just to get the numbers to add up); and	(ii) We have described a hypothetical scenario where assortativity preservation may be important—a setting with very high TB concentration in the elderly

(China). This illustrates why a particular update method (e.g., M2) might be considered desirable in particular demographic and epidemic context **(We have added this in the Introduction - lines 111–118**):

- *However, corrections to ensure reciprocity may alter underlying properties of the matrix, including assortativity (the relative preference of one group for contact with another) and the overall population-wide average contact rate. Depending on the specific research question, modellers may wish to preserve some or all these properties. For example, current evidence suggests that targeting TB vaccines to the elderly (in whom TB burden is concentrated) in China is most likely to be impactful as its population continues to age. Under these circumstances, failure to preserve contact assortativity may bias the estimates of elderly targeted vaccination.*

iii. potential implications of the specific modelling choices, especially in the context of vaccines.

(iii) We have clarified why ensuring reciprocity (M1) is essential and how a lack of reciprocity might affect age-specific force of infection (**We have added this in the Introduction - lines 100–103**). We also reinforced this in the example (above) where assortativity preservation (M2) may be required:

- *If total contacts between age groups are unbalanced, this leads to an error in the calculated age-specific force of infection parameters within the model. This propagates into errors in age-specific burden and introduces further error into the estimated impact of age-targeted interventions.*
-

As it stands, it feels more like an extended sensitivity analysis, attempting to cover all bases -- but if framed better, it could get at important questions at the heart of the matter about what empirical data are telling us, and why they matter.

We hope these provide the requested motivation for why and how these specific features about translating empirical data on contact matrices matter.

2. Why POLYMOD? Authors justify that India-specific data were not available in a fully representative form (though arguably a state within India is more representative that European nations), but why not data from a similarly densely populated China or has household structures that are more in line with Kenya? The specific choice of contact matrix data may not matter for the main question, but I think it worth authors addressing this choice.

We thank the reviewer for highlighting this important issue. In summary, we used POLYMOD data due to three factors:

(i) POLYMOD contains large, high quality, nationally representative data, whereas the currently available India-specific or India-like empirical data were from smaller, non-nationally representative, communities and not published in a form we could use in our study; (ii) the overall aggregate assortativity patterns of these smaller surveys or synthetic estimates were very similar to the POLYMOD data; and (iii) post hoc adjustment (following reviewer feedback) of the POLYMOD data for household, classroom, and workplace structures in India and into the age-groups used in our model led to a very similar matrix to an unadjusted POLYMOD matrix.

Especially due to factors (ii) and (iii), it is unlikely that using an alternative base contact matrix would alter our conclusions.

We give more details supporting our summary below.

1. To date, there are no nationally representative empirically derived contact data for India. Existing surveys are from smaller, non-nationally representative, communities (e.g., Kumar et al. [1]) or synthetic (Prem et al. [2]).
   Raw data of a form suitable to construct matrices for our model are not published for subnational surveys from other settings, e.g., by Kumar et al. (India) [1] or Kiti et al. (Kenya) [3]. The contact estimates published by Read et al. (China) [4] focus on differential contact patterns by level of urbanisation and show a broadly similar pattern in total contact rate and duration by age, albeit of a slightly greater magnitude, to POLYMOD. Our work does not stratify by location or urbanisation, so we could not incorporate this heterogeneity. **Nonetheless, this may serve as an important avenue warranting further work, so we have added this point to the manuscript discussion (below; lines 414–425).**

2. The assortativity patterns seen in these smaller/synthetic surveys was similar to the POLYMOD matrix we used, particularly at our level of age-group aggregation. **Points (1) and (2) are specifically addressed in the manuscript discussion as follows (lines 414–425):**
   Additionally, our use of a generic POLYMOD contact matrix rather than an India-specific matrix may reduce the accuracy of our findings. We know of two previous studies which estimated social contact patterns in India. Prem et al. [3] combined Demographic and Health Survey results, POLYMOD data, and other household-level data to generate synthetic age-specific contact matrices for India. Kumar et al. [7] reported a social contact survey limited to Haryana, North India. At the time of writing, raw data from neither study was available in the form needed to generate matrices for our model. However, similarly to the POLYMOD matrix, both studies found strong assortativity for in-age-group contacts, with additional assortativity between younger adults and children. Therefore, we speculate that differential impact across update methods is likely to remain stable, despite possible different magnitudes of vaccine impact and age-specific impact patterns.

3. In response to reviewer feedback, post hoc, we analysed whether the POLYMOD contact matrix, used at our level of age-group aggregation, was likely biased by differences in household structure, classroom, and workplace composition between India and the POLYMOD countries. In July 2021, after the completion of our study, Prem et al. published updated estimates of synthetic contact matrices for 177 countries [5], including India. These matrices were derived by adjusting the POLYMOD data with additional country-level data on sociodemographics, household structure, employment and school attendance (from the UN World Population Prospects, DHS and other sources). We used the total raw contacts predicted for India by Prem et al. to generate a three-age class matrix equivalent to our study. The resulting matrix (included in the technical appendix §6) was very similar to the

(unadapted) POLYMOD matrix used in our study. This analysis suggests that while the household structure in India will likely differ from POLYMOD countries, at nationally aggregated levels with the age groups used in our study, these differences are unlikely to bias our results substantially.

We have now specifically included this analysis in the manuscript as follows:

a. **Methods Lines 235–244:** *Country Adapted Matrix*
In July 2021, Prem et al [21] published an updated set of synthetic contact matrices for 177 countries derived by adapting POLYMOD data to reflect country-level demo-graphic, household, and location-specific composition (home, work, school, other) characteristics using UN World Population Prospects demographic projections, Demo-graphic and Health Survey data, and other sources. We used the estimated number of total contacts predicted for India by Prem et al to generate an equivalent matrix to Fig 1A. We compared this back-calculated matrix to that in Fig 1A to assess if country-specific adjustments led to substantially different contact rates or assortativity at this level of age-group aggregation.

b. **Results Lines 334–336:** *Finally, we found that adjustment for India-specific sociodemographic features did not lead to a substantially different base contact matrix compared to Fig 1A (appendix §6) at this level of aggregation of age-groups.*

c. **Discussion Lines 425–430**: *Furthermore, we found that adjustment for different household structure, classroom, and workplace composition did not substantially change the assortativity patterns of our base contact matrix. This analysis suggests that while household structure in India will likely differ from POLYMOD countries, at nationally aggregated levels with the age groups used in our study, these differences are unlikely to substantially bias our results.*

3. To what degree are diary-based contact patterns applicable to TB, where the transmission is airborne?
This is a general comment on the use of these data -- I would appreciate if the authors could provide their take on this: to what degree this is a data limitation (and these are the best data we have), or whether they believe that these data are fairly good representation of the age-specific contact patterns.

This is an important question. As Mtb is airborne, transmission can occur between individuals who “share air” but do not come into close contact [9,10]. Thus contact structures based on close contact may underestimate the pool of effective (potentially infectious) contacts. **We have reinforced this point and current data relating close vs casual contact in the manuscript discussion as follows (lines 450–462):**

- *Most empirical contact studies—including POLYMOD—report rates of “close contact”, i.e., a social contact involving physical touch or sustained conversation. Close contacts are likely to represent those required for transmission of direct contact or droplet infection. However, Mtb transmission is airborne; the pool of potentially infectious contacts is likely to be larger and include, for example, individuals who ‘share the same air’ in poorly ventilated spaces. However, contact data for such ‘casual contacts’ is very sparse. Data from South Africa report that age-assortativity patterns in the number of*

contacts is similar between ‘close’ and ‘casual’ contacts, but contact time is more age-assortative in ‘casual’ contacts. If this finding holds in settings outside of South Africa, then out study—which uses contact rates but not time—is less likely to be biased by using ‘close contact’-based data. However, if further empirical data on ‘casual contacts’ or their differences with ‘close contacts’ emerges, this would be a useful avenue for further research.

4. Are the results generalizable? I think it would be great if the authors could speak to:
   i. whether their finding (no significant difference) is tied to the choice of contact matrix (also relates to point 2), and/or
   ii. the projected demographic changes in India.

One suggestion here, if logistically possible, would be to conduct sensitivity analyses with one of the alternative contact matrix data and or demographic changes.

We thank the reviewer for this comment. We cannot definitively comment on the generalisability of our finding but hypothesise that the projected demographic change combined with the age-specific pattern of TB likely leads to the lack of observed difference.

We have clarified this in the manuscript discussion (**lines 463–473**) as:

- *In the Indian context modelled here, it is likely that projected demographic change combined with the age-specific pattern of TB likely contributed to the lack of observed difference in vaccine impact. The Indian population is projected to age, with an increased fraction of elderly compensated by a reduction in children. The adult group, responsible for most Mtb transmission and burden, remains relatively stable, both in absolute and fractional terms. As such, the specific changes to children and elderly fractions, though substantial, had smaller impact on the TB epidemic and therefore vaccine impact. Further work is required to investigate whether our findings are generalisable in particular in settings with more significant changes to demographic composition, with different patterns of age-specific disease burden, and with diseases with shorter time courses.*

Minor points.
Suggest specifying age-specific contact structure, since contact structure can be based on other non-age related factors
Fig 1: Suggest spelling out C, A, and E.
Introduction, lines 62-65: Please include references: it will also make help make authors point more clear.

Thank you. These points have been addressed in the manuscript.

**Reviewer 3**	
The authors investigated the effects of updating age-specific contact matrices to match the changing demography on the estimates for vaccination impact. The subject of the paper is interesting and important, and the paper is overall well written.	**Please note all line numbers are with "No Markup" enabled in the Track Changes mode.**<br>We thank the reviewer for their kind feedback.
1. I think it helps to clearly define upfront what a "contact matrix" refers to. Does it represent the average (daily) number of contacts that an individual in age group i makes with an individual in age group j? Does it represent the (daily) number of contacts between the members of age groups i and j (in which case, the matrix should be symmetrical).	Thank you. Conventionally (and in our study), the entries of a contact matrix denote the average daily number of contacts that each member of some age group *i* has with members of *j* (and each other age group). **We have clarified this in the manuscript introduction (lines 69–71).**<br>● *These matrices present the number of contacts that each member of some age group i makes with members of each age group in the model over a defined time period (typically daily).*
2. Page 8, lines 154-156: Would you please provide the details of how the contact matrices of Fig 1A are generated from the raw POLYMOD contact data?	Thank you. **We have clarified this in the methods section (lines 177–185) as follow**s:<br>● *Data from POLYMOD are expressed as the average number of contacts made per day by each survey participant per country, with participants binned into 5-year age groups. We used this data and the population size of the participant age group to generate the total contacts made by all participants in that age group. This was then aggregated into the specific groups in our model (0–14, 15–64, and 65+). Finally, totals were summed across all POLYMOD countries, then divided by total population to generate POLYMOD-wide average daily contact ratesWe aggregated the raw POLYMOD contact data and used the demographic structure of the constituent countries to generate a three age-group base matrix (Fig 1A) that corresponded to a snapshot of social contact at the time of the survey (2005–2006).*
3. Page 10, lines 195-204: It is not clear why β (the scaled per-capita effective contact rate) is introduced. It might make sense to move the mathematical definition of β from the supplement to the main text. Also, in page 6 of the supplement, β seems to be representing both a matrix and a scalar.	Thank you. We introduce the scaled per-capita effective contact rate to aid the reader when; comparing contact rate trends between update methods M0–M3.<br>In the M3 update, the contact matrix is normalised by dividing each entry by the population-wide mean contact rate. Consequently, the magnitude of matrix entries becomes smaller than entries in the M0–M2 matrices, which would be reflected in a direct comparison of the contact rates (κ) between update methods. However, the

	sampled values for the probability of transmission per contact (π) are higher for M3, compensating for this reduction, leading β values of comparable magnitude.<br><br>**We have moved the full expression for β into the main text (line 230). Additionally, the matrix containing individual pairwise β terms has been renamed B in the appendix.**
Minor issues:<br>4. Page 6 of the supplement: at the last paragraph, the references to figures missing.<br>5. Page 7 of the supplement: It is not clear what "contact matrix2.2" is referring to.	Thank you. These have been corrected.

References

1. Kumar S, Gosain M, Sharma H, Swetts E, Amarchand R, Kumar R, et al. Who interacts with whom? Social mixing insights from a rural population in India. PloS One. 2018;13: e0209039. doi:10/gh58j5
2. Prem K, Cook AR, Jit M. Projecting social contact matrices in 152 countries using contact surveys and demographic data. PLOS Comput Biol. 2017;13: e1005697. doi:10/gbwvz2
3. Kiti MC, Kinyanjui TM, Koech DC, Munywoki PK, Medley GF, Nokes DJ. Quantifying age-related rates of social contact using diaries in a rural coastal population of Kenya. PloS One. 2014;9: e104786. doi:10/gmms22
4. Read JM, Lessler J, Riley S, Wang S, Tan LJ, Kwok KO, et al. Social mixing patterns in rural and urban areas of southern China. Proc R Soc Lond B Biol Sci. 2014;281: 20140268. doi:10/gf3mp5
5. Prem K, Zandvoort K van, Klepac P, Eggo RM, Davies NG, Group C for the MM of IDC-19 W, et al. Projecting contact matrices in 177 geographical regions: An update and comparison with empirical data for the COVID-19 era. PLOS Comput Biol. 2021;17: e1009098. doi:10/gmms24
6. Harris RC, Sumner T, Knight GM, Evans T, Cardenas V, Chen C, et al. Age-targeted tuberculosis vaccination in China and implications for vaccine development: a modelling study. Lancet Glob Health. 2019;7: e209–e218. doi:10/ghk49b
7. Harris RC, Sumner T, Knight GM, Zhang H, White RG. Potential impact of tuberculosis vaccines in China, South Africa, and India. Sci Transl Med. 2020;12. doi:10/ghk48d
8. Weerasuriya CK, Harris RC, McQuaid CF, Bozzani F, Ruan Y, Li R, et al. The epidemiologic impact and cost-effectiveness of new tuberculosis vaccines on multidrug-resistant tuberculosis in India and China. BMC Med. 2021;19: 60. doi:10/gh56v3
9. McCreesh N, Morrow C, Middelkoop K, Wood R, White RG. Estimating age-mixing patterns relevant for the transmission of airborne infections. Epidemics. 2019;28: 100339. doi:10/ggvp8w
10. Raffalli J, Sepkowitz KA, Armstrong D. Community-based outbreaks of tuberculosis. Arch Intern Med. 1996;156: 1053–1060.
