# Supplementary material for: Updating age-specific contact structures to match evolving demography in a dynamic mathematical model of tuberculosis vaccination
Source: PLoS Comput Biol. 2022 Apr 22;18(4):e1010002. doi: 10.1371/journal.pcbi.1010002 (PMC9067655; doi:10.1371/journal.pcbi.1010002)
Supplement: Attachment — Submitted filename: PCOMPBIOL-D-21-01594_R2_Response to reviewers.pdf [file pcbi.1010002.s004.pdf]

TB Modelling Group and TB Centre,
Department for Infectious Disease Epidemiology
London School of Hygiene & Tropical Medicine
Keppel Street, London WC1E 7HT
+44 (0)20 7636 8636 • www.lshtm.ac.uk

Feb 28, 2022

Re: PCOMPBIOL-D-21-01594 — Updating Age-specific Contact Structures to Match Evolving Demography in a Dynamic Mathematical Model of Tuberculosis Vaccination

Dear Editors,

We thank you and the reviewers for reviewing the changes to the manuscript and your favourable comments.

Please find attached the edited manuscript, incorporating the three suggestions from reviewer 2, as follows:

1. Suggest including "age-specific" in the title.	Thank you. Added.
2. line 94, "interact with" is written twice	Thank you. Deleted.
3. Suggest including a caveat that although these specific results were not affected, including county-specific contact patterns could be important for general transmission modeling with age-specific contact patterns, just as incorporating evolving demographic changes.	Thank you. We have added the following:<br><br>**[Line 474–479 Discussion - track changes version]**<br><br>*Finally, in this study, we investigated whether varying methods of updating contact matrices to match demography differentially affected vaccine impact estimates. However, for studies that aim to accurately estimate disease burden or intervention impact, country-specific contact structures grounded in data will likely be important to accurately characterise age-specific mixing.*

We hope these revisions adequately address the remaining reviewer suggestions.

In addition, we have corrected some minor typographical errors and made some small edits to improve readability. These are indicated in track changes.

Please do not hesitate to let us know if any further clarification is required. We look forward to your correspondence.

Thank you and best wishes,

Chathika Weerasuriya
On behalf of all authors
